# Untangling the brain's neuroinflammatory and neurodegenerative transcriptional responses

**DOI:** 10.1038/ncomms11295

**Published:** 2016-04-21

**Authors:** Karpagam Srinivasan, Brad A. Friedman, Jessica L. Larson, Benjamin E. Lauffer, Leonard D. Goldstein, Laurie L. Appling, Jovencio Borneo, Chungkee Poon, Terence Ho, Fang Cai, Pascal Steiner, Marcel P. van der Brug, Zora Modrusan, Joshua S. Kaminker, David V. Hansen

**Affiliations:** 1 Department of Neuroscience, Genentech, Inc., 1 DNA Way, South San Francisco, California 94080, USA; 2 Department of Bioinformatics, Genentech, Inc., 1 DNA Way, South San Francisco, California 94080, USA; 3 Department of Molecular Biology, Genentech, Inc., 1 DNA Way, South San Francisco, California 94080, USA; 4 Department of Immunology, Genentech, Inc., 1 DNA Way, South San Francisco, California 94080, USA; 5 Department of Diagnostics, Genentech, Inc., 1 DNA Way, South San Francisco, California 94080, USA

## Abstract

A common approach to understanding neurodegenerative disease is comparing gene expression in diseased versus healthy tissues. We illustrate that expression profiles derived from whole tissue RNA highly reflect the degenerating tissues' altered cellular composition, not necessarily transcriptional regulation. To accurately understand transcriptional changes that accompany neuropathology, we acutely purify neurons, astrocytes and microglia from single adult mouse brains and analyse their transcriptomes by RNA sequencing. Using peripheral endotoxemia to establish the method, we reveal highly specific transcriptional responses and altered RNA processing in each cell type, with Tnfr1 required for the astrocytic response. Extending the method to an Alzheimer's disease model, we confirm that transcriptomic changes observed in whole tissue are driven primarily by cell type composition, not transcriptional regulation, and identify hundreds of cell type-specific changes undetected in whole tissue RNA. Applying similar methods to additional models and patient tissues will transform our understanding of aberrant gene expression in neurological disease.

## Abstract (web summary)

Whole tissue RNA profiling can help identify altered molecular pathways underlying neurodegenerative disease, but often masks cell type-specific transcriptional changes. Here, the authors compare transcriptomes of neurons, astrocytes, and microglia from Alzheimer's disease model brains and identify hundreds of cell-type specific changes.

## Graphical abstract

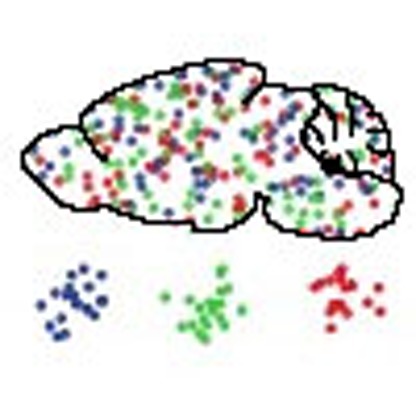

One approach to better understand the molecular mechanisms of neurodegenerative disease is to compare gene expression profiles from diseased versus control tissues and draw inferences about which biological pathways and cellular processes are altered in the disease state. However, the cellular complexity of central nervous system (CNS) tissue, in which glial cell types including microglia and astrocytes are interspersed among neurons of many subtypes, limits the utility of this approach. Expression profiles derived from whole tissue RNA represent each gene's average expression among all cells but do not reveal which cell types are responsible for a gene's normal or altered expression in healthy or diseased tissues. Lacking such information, the genes and pathways implicated by profiling whole tissues cannot be readily incorporated into cellular models of neurodegenerative disease. Moreover, changes in a gene's expression that occur in a specific cell type may be undetected in whole tissue RNA as the difference may be masked by the overall signal from all cell types.

To circumvent these shortcomings, researchers have developed methods to acutely isolate individual cell types from adult brain tissue. Most commonly, brain tissue is dissociated into single cells, from which microglial/macrophage-type cells—specifically labelled genetically (for example, *Cx3cr1::GFP* expression) or biochemically (for example, anti-CD11b)—are purified by fluorescence-activated cell sorting (FACS) or other antibody-based methods^1^,^2^. Using similar methods, researchers have also isolated astrocytes, neurons, endothelial cells and other brain cell types^3^,^4^,^5^,^6^, yet these significant advances have certain limitations. First, most dissociation methods involve enzymatic treatment at warm or ambient temperatures^1^,^7^,^8^,^9^, allowing stress-induced changes in RNA profiles to occur throughout the procedure. Second, genetic labelling methods require extra resources and time to obtain the desired cell type labelled at the appropriate disease stage and in the proper genetic background, and may also interfere with normal biology^10^,^11^. Third, researchers often focus on a cell type of particular interest rather than study multiple cell types from the same brain, so correlative cell type analyses within specimens cannot be performed. Fourth, samples are often pooled to increase RNA yield and detection, obscuring animal-to-animal variability and increasing the required number of specimens. Fifth, many gene expression studies have used microarrays or other technologies that are becoming outmoded by the advent of high-throughput RNA sequencing, which has enabled more comprehensive transcriptomic analyses.

Here we utilize an approach that avoids some of the above-mentioned limitations^12^ and adapt it further to isolate populations of neurons, astrocytes and microglia from single adult brain specimens and analyse their transcriptomes by RNA amplification and sequencing. To our knowledge, this is the first report of these three cell populations being purified simultaneously from the brain of an adult mouse and analysed by RNA sequencing (RNA-Seq). The method does not require incubations at warm temperatures for enzymatic dissociation, genetic labelling of any cell type or pooling of samples. Using peripheral endotoxemia as an acute neuroinflammatory model to establish the method's utility, we demonstrate the diversity and specificity of each cell type's transcriptional and RNA processing responses. We observe correlations in animal-to-animal variability between cell types and investigate the tumour-necrosis factor (TNF) pathway's contribution to the brain's endotoxemia response.

We also use cell type-specific sequencing data to probe existing data sets of gene expression in neurodegenerative disease tissues from human patients and/or animal models of frontotemporal dementia (FTD), amyotrophic lateral sclerosis (ALS) and Alzheimer's disease (AD). We provide evidence that disease-related changes in expression profiles from whole tissue RNA are often not due to transcriptional regulation but rather the altered cell type composition of disease tissue samples. Finally, we apply our sorting method to a mouse model of AD to reveal many pathology-related transcriptional changes in purified cell types that were not observed in whole tissue RNA.

## Results

### Altered cellularity in neurodegenerative expression profiles

To better understand existing data sets for gene expression in neurodegenerative diseases, we used recent RNA-Seq data from different cell types acutely isolated from postnatal mouse brains^9^ to predict which cell types were most likely responsible for disease-related changes observed in whole tissue. We also used our own RNA-Seq data from neurons, astrocytes and microglia acutely isolated from adult mouse brain (see next section for details) to further inform the results.

**Figure 1:**
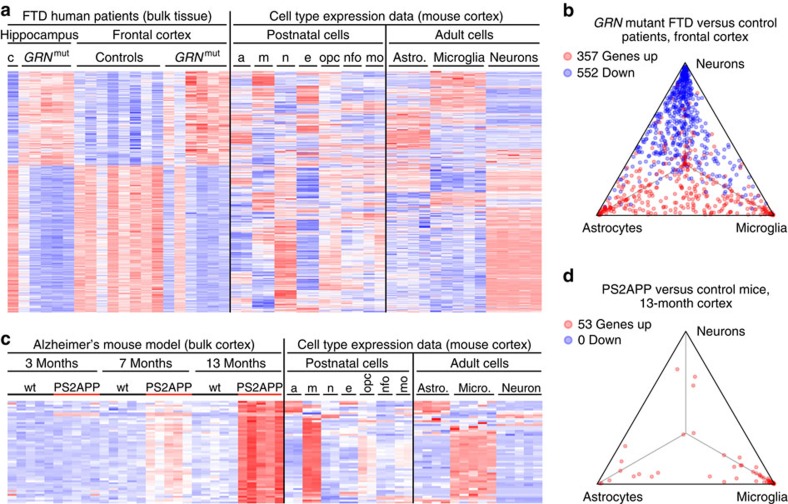
Mapping dementia-related gene expression profiles to CNS cell types. (**a**) Genes differentially expressed by microarray analysis (fold change ⩾2, adjusted *P*≤0.05) in *GRN* mutant FTD patient cortex versus controls (GSE13162, imported) were analysed for expression in cerebrocortical cell types from normal postnatal (GSE52564, imported) or adult (GSE75246, this study) mouse brains. Each gene's expression was *Z*-score normalized separately within each of the three data sets (colour range: −4.10≤Z≤5.23). Rows (genes) were clustered hierarchically. Columns (RNA samples) were sorted by sample metadata. (**b**) The same genes with higher (red) or lower (blue) expression in *GRN* mutant FTD cortex are plotted in this triangle. A gene's proximity to a corner represents its degree of preferential expression in the indicated cell type from normal adult mouse cortex (see the Methods for details). (**c**,**d**) Similar to **a**,**b**, this heat map (colour range: −2.20≤Z≤3.47) and triangle plot analyse cell type expression for genes differentially expressed by microarray in bulk cerebral cortex from the PS2APP Alzheimer's model versus wild-type mice at 13 months of age (GSE74995, this study). a/astro., astrocytes; c, control; e, endothelial cells; m/micro., microglia; mo, myelinating oligodendrocytes; n, neurons; nfo, newly formed oligodendrocytes; opc, oligodendrocyte progenitor cells; wt, wild type.

We first looked at several hundred genes whose expression changed at least twofold (adjusted *P*≤0.05) in a microarray analysis of frontal cortex samples from FTD patients with mutations in the progranulin (*GRN*) gene, relative to non-diseased control samples^13^. When we mapped each gene to the cell type(s) most likely responsible for its expression, the overall trend was striking. Most of the transcripts with increased abundance in FTD cortex mapped to non-neuronal cell types—astrocytes and microglia, as well as endothelial cells—whereas most of the transcripts with decreased abundance mapped to neurons (Fig. 1a,b). This suggested that many of the changes in gene expression were influenced by the altered cellular composition of end stage FTD tissue, with extensive neuronal loss and prominent gliosis.

We next applied this type of analysis to our microarray data from the PS2APP transgenic AD model, in which mutant alleles of *APP* and *PSEN2* give rise to age-dependent amyloid plaque pathology^14^. In 13-month aged mice, 53 genes (including *Thy1* and *Prnp* transgene elements) showed ⩾2-fold increased expression (adjusted *P*≤0.05) in PS2APP versus non-transgenic cortex. Most of these differentially expressed genes were normally expressed by microglia, and some by astrocytes (Fig. 1c,d). Unlike in FTD cortex, we did not see ⩾2-fold downregulation of any neuronal genes (or any genes) in PS2APP cortex. These observations were consistent with histopathological features of PS2APP brains, including plaque-associated gliosis but no notable loss of neurons, suggesting that many instances of increased gene expression in PS2APP cortex were due, at least in part, to increased glial cell numbers rather than transcriptional upregulation.

**Figure 2:**
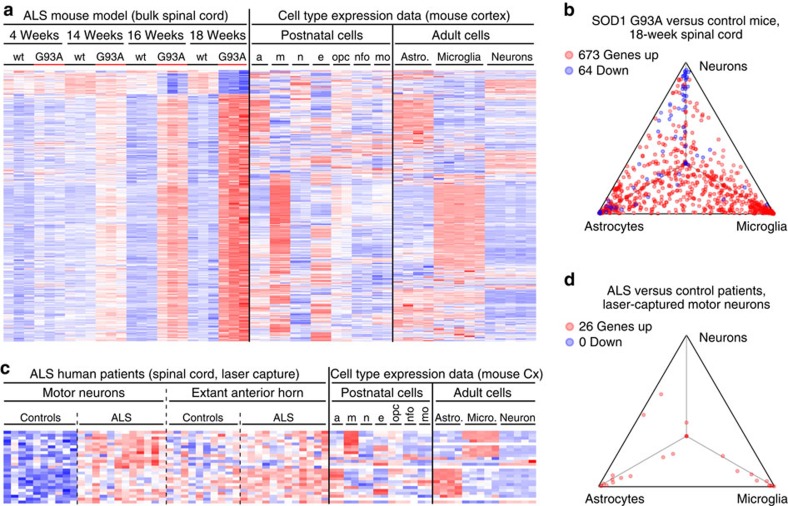
Mapping ALS-related gene expression profiles to CNS cell types. (**a**,**b**) Similar to Fig. 1a,b, this heat map (colour range: −3.34≤Z≤3.68) and triangle plot analyse cell type expression for genes differentially expressed in bulk spinal cords from the SOD1 G93A ALS model versus wild-type mice at 18 weeks of age (GSE18597, imported). (**c**,**d**) Similar to Fig. 1a,b, this heat map (colour range: −2.75≤Z≤3.14) and triangle plot analyse cell type expression for genes differentially expressed in laser-captured motor neuron samples from ALS patient spinal cords versus controls (GSE18920, imported). Expression data from anterior horn material remaining after motor neuron excision are also shown. a/astro., astrocytes; Cx, cortex; e, endothelial cells; m/micro., microglia; mo, myelinating oligodendrocytes; n, neurons; nfo, newly formed oligodendrocytes; opc, oligodendrocyte progenitor cells; wt, wild type.

Finally, we performed similar analyses on data sets from a murine ALS model and from ALS patients. RNA samples from intact spinal cords of mice expressing mutant human SOD1 (hSOD1^mut^) showed hundreds or dozens of genes whose expression was increased or decreased, respectively, at least twofold (adjusted *P*≤0.05) compared with samples from non-transgenic mice^15^. As with FTD samples, most of the transcripts with increased abundance in hSOD1^mut^ mice mapped to astrocytes and microglia, as well as endothelial cells, whereas the majority of transcripts with decreased abundance mapped to neurons (Fig. 2a,b)—likely reflecting the gliosis and neuronal loss that progressively occur with ageing in hSOD1^mut^ spinal cords. Consistent with this interpretation, most expression differences between hSOD1^mut^ and non-transgenic spinal cords magnified as the mice aged (Fig. 2a). Turning our attention to the human disease, we examined gene expression data from laser-captured motor neurons microdissected from spinal cords of ALS or control patients^16^. Surprisingly, most of the 26 genes with ⩾2-fold increased RNA abundance (adjusted *P*≤0.05) in ALS motor neuron samples were predicted to be expressed in microglia or astrocytes rather than in neurons (Fig. 2c,d). Thus, despite meticulous efforts to identify transcriptional changes in ALS motor neurons, most of the observed differences may have derived from contaminating gliotic cells, or fragments thereof, co-excised with the neurons. Moreover, no genes met the cutoff of ⩾2-fold decreased expression in human ALS motor neuron samples, further implying that depletion of certain neuronal RNAs in hSOD1^mut^ spinal cords reflects neuronal loss, whereas gene expression in extant motor neurons is relatively unchanged.

**Figure 3:**
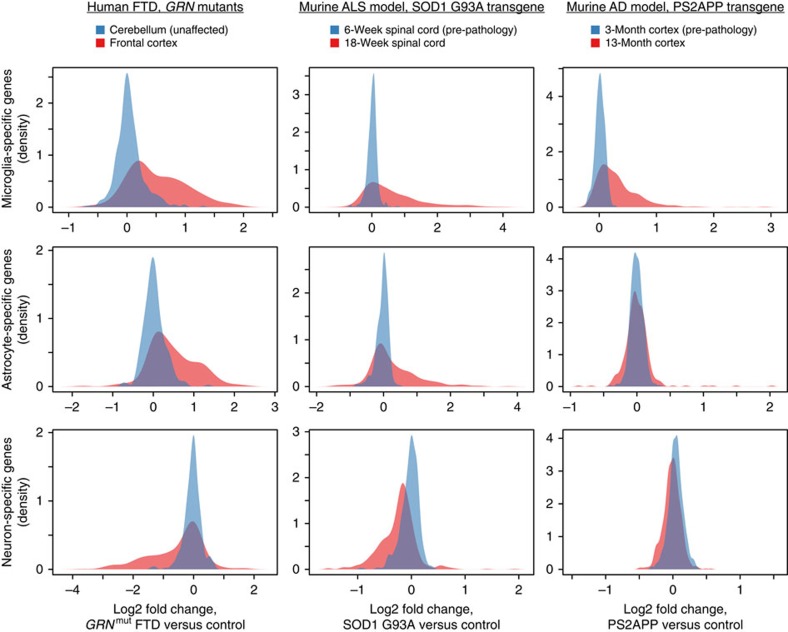
Biased expression of cell type-specific genes in neurodegenerative tissues. Genes specifically expressed by microglia, astrocytes or neurons were selected as those with ⩾20-fold enrichment compared with the other two cell types when isolated from normal adult mouse cortex and analysed by RNA-seq. For the subsets of these genes or their human homologues that were probed by microarray, distributions are shown for fold-changes in bulk cortex from FTD patients, bulk spinal cord from murine ALS model or bulk cortex from murine AD model, relative to controls, in unaffected (blue) and affected (red) regions and times. The shift of a red distribution to the right is consistent with higher content of that cell type in the affected tissue (for example, microglia in AD model and astrocytes in ALS model), whereas a shift to the left suggests a depletion of that cell type (for example, neurons in FTD cortex).

To further explore how cellular composition may influence neurodegenerative disease transcriptional profiles, we took a complementary approach by looking at whether RNAs specifically expressed by microglia or astrocytes in normal tissue showed overall enrichment in disease tissues, and whether neuron-specific RNAs showed overall depletion (Fig. 3). Microglia-specific RNAs were indeed enriched in cortex from *GRN* mutant FTD patients, spinal cord from hSOD1^mut^ mice and cortex from PS2APP mice. Astrocyte-specific RNAs were similarly enriched in human FTD cortex and mouse hSOD1^mut^ spinal cord, but not in mouse PS2APP cortex. In contrast, neuron-specific RNAs were clearly depleted in FTD cortex and hSOD1^mut^ spinal cord, whereas depletion was minimal in PS2APP cortex. These observations were perfectly consistent with known histopathology in these tissues—neuronal loss and gliosis in FTD and hSOD1^mut^-driven ALS models, and gliosis, but little if any neuronal loss, in amyloid-based AD models. The astrogliosis observed in AD models and in human AD tissue involves morphological changes but not cellular proliferation, whereas microglia clearly proliferate^17^,^18^. Expression profiles in neurodegenerative disease tissues are thus considerably influenced by altered cellular composition, and profiles from whole tissue RNA may be misleading if changes in disease are assumed to result from transcriptional modulation.

### Purifying neurons, astrocytes and microglia from adult brain

**Figure 4:**
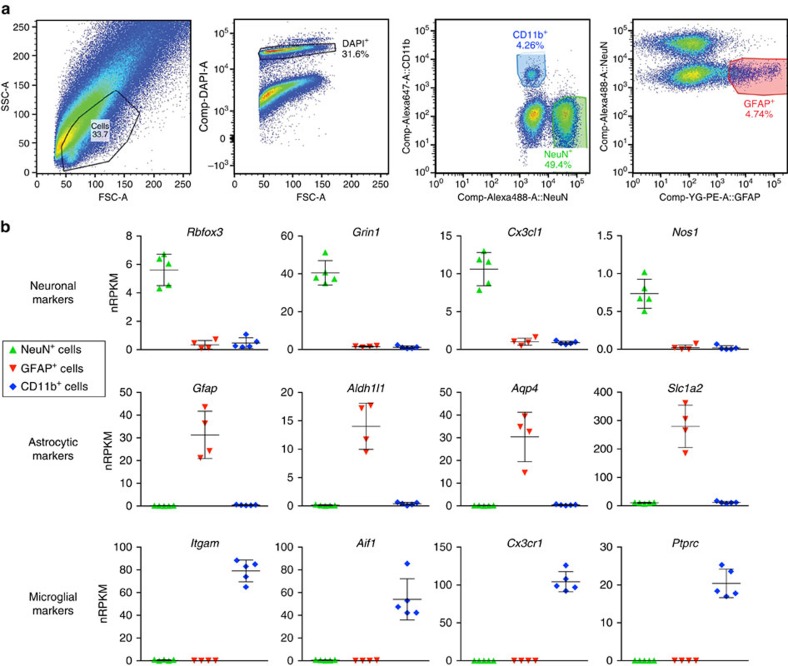
Antibody-based FACS and RNA-Seq for CNS cell types from adult brain tissue. (**a**) Left panel shows forward/side scatter (FSC/SSC) of dissociated cortical tissue. DAPI^+^ events (‘cells') were gated to select for nuclei-containing singlets (second panel), and neurons and microglia were isolated as two distinct populations corresponding to NeuN^+^CD11b^–^ and NeuN^−^CD11b^+^ cells, respectively (third panel). The NeuN^−^ population was further used to isolate GFAP^+^ astrocytes (last panel). (**b**) RNA-Seq data confirmed that NeuN^+^ sorting enriched for cells expressing neuronal markers, GFAP^+^ sorting enriched for cells expressing astrocytic markers, and CD11b^+^ sorting enriched for cells expressing microglial markers (*n*=5 animals; one astrocyte sample was excluded from the analysis due to evidence of neuronal contamination). Bars represent mean±s.d. (Prism).

The above observations illustrate the need to isolate specific cell types in order to understand transcriptional changes in diseased or injured CNS tissue. Using the method of Guez-Barber *et al*.^12^ for purifying neurons or endothelial cells from rat brain as a starting point, we set out to dissociate and immunopurify cortical neurons, astrocytes and microglia from a single adult mouse brain, with no genetic cell type labelling, for analysis by RNA sequencing. To avoid stress-related transcriptional responses that occur during warm enzymatic incubations, we performed all procedures at 4 °C or on ice, including Accutase treatment for 30 min followed by mechanical dissociation. The suspension was filtered (70 μm) and then centrifuged through Percoll to enrich for cells and reduce debris. The pelleted fraction was resuspended, subjected to mild ethanol fixation and immunolabelled using anti-CD11b to label microglia, anti-glial fibrillary acidic protein (GFAP) to label astrocytes, and anti-NeuN to label neurons. Cell populations were collected by FACS (Fig. 4a), followed by RNA extraction from purified cell types.

The RNA levels for the immunolabelling targets CD11b (*Itgam*), GFAP and NeuN (*Rbfox3*) were appropriately enriched or depleted in each sorted cell population when analysed by reverse transcription–quantitative PCR (RT–qPCR; Supplementary Fig. 1a). To verify appropriate cell type enrichment in sorted populations, we also analysed additional RNAs encoding the cell type markers Cx3cr1, Iba1 (*Aif1*) and CD45 (*Ptprc*) for microglia; Aldh1l1, Aqp4 and GLT1 (*Slc1a2*) for astrocytes; and Grin1, Cx3cl1 and nNOS (*Nos1*) for neurons. RNA for each marker was enriched in the expected cell population relative to the other two populations (Supplementary Fig. 1b), confirming the successful purification of microglia, astrocytes and neurons from adult cortex.

### Cellular specificity of CNS endotoxic transcriptional response

To test the method's suitability for detecting treatment-dependent, genome-wide, transcriptional changes within each cell type, we injected mice peripherally with saline or with the endotoxin lipopolysaccharide (LPS), which induces an inflammatory response in the CNS^19^,^20^,^21^. One day post injection, we dissociated and FACS-purified cell populations from perfused cortex, followed by RNA purification, amplification and sequencing analysis. RNA-Seq data showed appropriate marker enrichment in sorted cell populations (Fig. 4b), confirming our earlier RT–qPCR data. The expression profiles we obtained for microglia, astrocytes and neurons from control adult mice were generally similar to profiles reported elsewhere for the same cell types isolated from postnatal mouse brains using different methods^9^ (see Supplementary Data 1 for details). However, our data set may better reflect unperturbed microglia, as certain LPS-induced genes in our study including *Cst7*, *Cd300lf* and *Ccl5* were expressed only at background levels (similar to neurons) in microglia from saline-injected mice but showed high microglia-specific expression in the data set of Zhang *et al*.^9^ (Supplementary Fig. 2a), possibly due to transcriptional induction during their enzymatic treatment of tissues at warm temperature. In addition, our neuron expression data reflect mature rather than immature stages of development. Thus, certain genes highly expressed in adult but not fetal human cortex^22^ show neuron-specific expression in our data from adult mice but not in the data of Zhang *et al*. from postnatal mice. Conversely, certain genes highly expressed in fetal but not adult human cortex show neuron-specific expression in the data of Zhang *et al*., but not in our adult data (Supplementary Fig. 2b).

**Figure 5:**
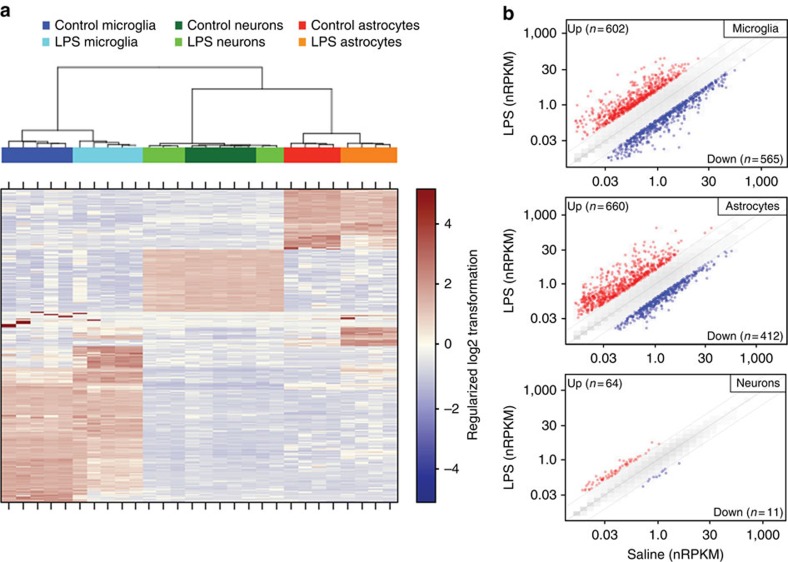
Genome-wide, cell type-specific expression profiles of the brain's endotoxic transcriptional response. (**a**) We performed unsupervised clustering of sorted brain cell RNA-Seq data from saline- and LPS-injected mice (*n*=5 animals per treatment) using the 2.5% most variably expressed genes across all samples. The samples segregated into distinct clusters determined primarily by lineage and cell type. Microglia and astrocyte samples further segregated according to treatment. (One astrocyte sample from each group was excluded from the analysis due to evidence of neuronal contamination.) (**b**) Genome-wide ‘two-way' plots of average gene expression levels in microglia, astrocytes or neurons isolated from LPS-injected versus saline-injected mice. Genes whose average expression increased (red) or decreased (blue) at least fourfold (adjusted *P*≤0.05, Wald test, DESeq2) are plotted.

Unsupervised clustering of our samples using the 2.5% most variably expressed genes segregated the samples into distinct groups according to cell type (Fig. 5a). The peripheral endotoxemia had a profound effect on gene expression in both microglia and astrocytes, as samples within these cell types clustered naturally into treatment and control groups. Neuron samples did not cluster into separate treatment and control groups at this level of analysis, indicating that the brain's most pronounced transcriptional responses to endotoxemia occurred within glial cell types. Next, we plotted the mean expression level for every gene in each cell type from LPS-injected versus saline-injected mice (Fig. 5b; see also Supplementary Data 2 for details). This further illustrated the neurons' relative lack of transcriptional response, in contrast to glial cells. Microglia and astrocytes each showed more than a thousand genes with ⩾4-fold change in expression (adjusted *P*≤0.05), compared with only 75 such changes in neurons.

**Figure 6:**
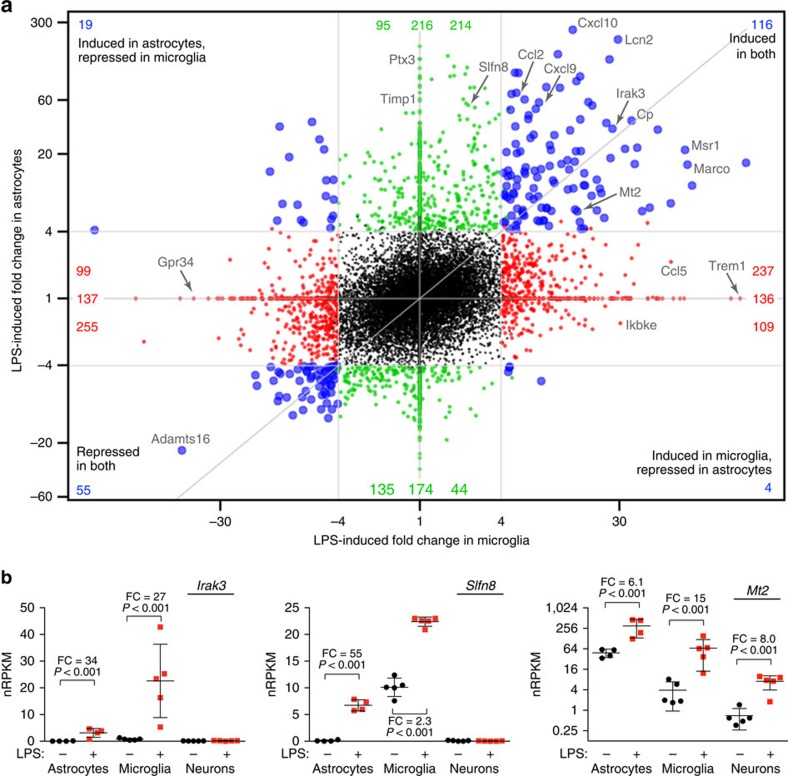
Distinct transcriptional responses to endotoxemia in microglia and astrocytes. (**a**) Genome-wide ‘four-way' plot for every gene's LPS-induced fold change (FC) in astrocyte expression versus LPS-induced FC in microglial expression. Each coloured dot represents a gene that met the cutoffs of FC⩾4, adjusted *P*≤0.05 in microglia only (red), in astrocytes only (green) or in both cell types (blue). Higher induction in terms of FC should not be conflated with absolute expression levels (see examples in **b**). Genes below an expression threshold (see the Methods for details) in a cell type are plotted as FC=1 for that cell type. (**b**) Individual expression plots of RNA-Seq data for *Irak3*, *Slfn8* and *Mt2* genes in each cell type from saline- and LPS-injected mice (*n*=5 animals per treatment), with FC and adjusted *P*-values shown (Wald test, DESeq2). Bars represent mean±s.d. (calculated by Prism).

Microglia and astrocytes are both widely known to mediate CNS inflammatory signalling, but the endotoxic transcriptional responses in these cell types were remarkably distinct, with only ∼15% of the genes with altered expression in either cell type being similarly regulated in the other (Fig. 6a; see also Supplementary Data 2 to compare LPS responses of these cell types with that of neurons). Even among genes similarly affected in both cell types, some were primarily expressed by one cell type in terms of absolute expression level, further emphasizing cellular specificity in the brain's endotoxic transcriptional response (Fig. 6b). Compared with previous reports of astrocytic^3^ and microglial^23^ responses to endotoxemia, our data revealed hundreds of additional LPS-responsive genes in each cell type (Supplementary Figs 3 and 4).

An attractive prospect of this method is detecting changes within individual cell types that are masked in whole tissue RNA. To demonstrate this advantage and to replicate our initial findings using a different technology, we selected several LPS-responsive genes from our sorted cell RNA-Seq data that seemed unlikely to appear as LPS-responsive in whole tissue RNA because of constitutive expression in other cell types. We injected new groups of mice, and 24 h later purified whole tissue RNA from one hemicortex and individual cell types from the other. Testing the RNA samples by RT–qPCR validated our RNA-Seq data and verified our predictions, confirming that genes including *Gas7*, *Kcna3*, *Adora2a* and *Ppargc1b* were LPS-induced in microglia; that genes including *Nav1*, *Vipr1*, *St6gal1*, *Sema4g*, *Dock4* and *Plxna4* were LPS-repressed in microglia; and that all of these changes were obscured in whole tissue RNA (Supplementary Fig. 5). In other examples, *Arap3* and *Slc7a8* expression increased in neurons and astrocytes from LPS-injected mice but not in whole tissue, being offset by endotoxic repression in microglia (Supplementary Fig. 5).

Another advantage of this method is the ability to correlate expression in different cell types from the same animal. For example, we found that the animal LPS6, and to a lesser extent LPS5, showed a weak LPS response in multiple cell types (Supplementary Fig. 6). Thus, this method turns what would otherwise be isolated outliers into a consistent signal—animals in which the global LPS response, for whatever reason, was attenuated in multiple cell types.

### Brain RNA processing altered in endotoxemia

RNA biology is increasingly recognized as a potential contributor to the aetiology of neurological disease^24^. To demonstrate the feasibility of profiling differential RNA processing in CNS cell types, we used our RNA-Seq data to quantify the inclusion frequencies of annotated and novel RNA processing events^25^ and test for LPS-induced changes in those events^26^. To our knowledge, little is known about the effect of endotoxemia on RNA processing in the CNS.

**Figure 7:**
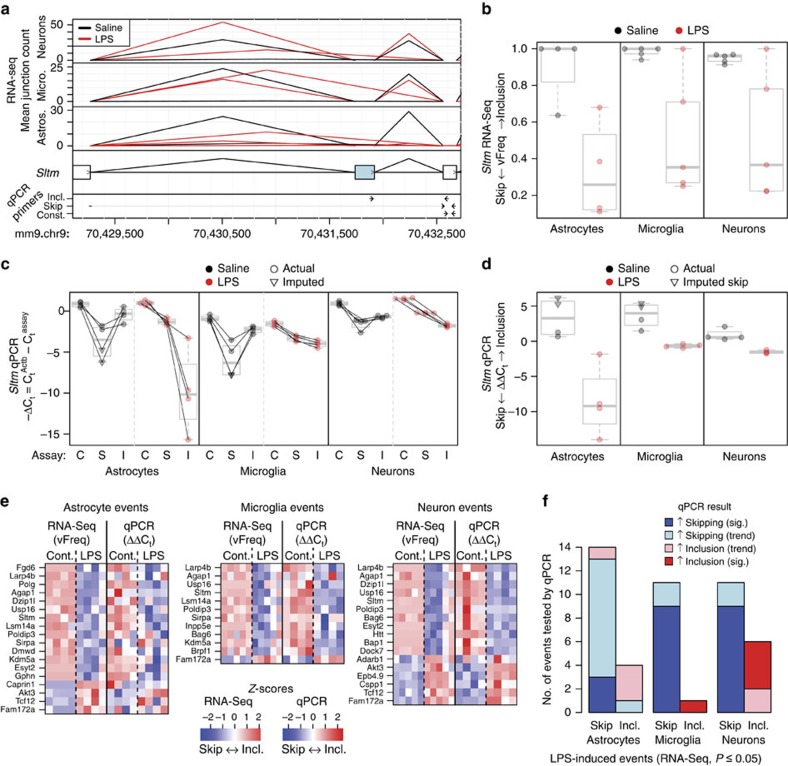
Endotoxemia-induced RNA splicing alterations in CNS cell types. (**a**) RNA-Seq junction reads for example event in *Sltm* gene. Peaks represent detected splice junctions with heights indicating average size factor-normalized coverage within each treatment group. The skipping junction (longer peak) is detected in all three cell types from LPS-treated mice but very low in the vehicle group. Three annotated *Sltm* exons (cassette exon in blue) and qPCR primers are shown below. Grey bar for the skipping assay indicates a junction-spanning primer. (**b**) RNA-Seq reads from same *Sltm* event summarized as variant frequencies (vFreq) between 0 (complete skipping) and 1 (complete inclusion). The single samples in the microglia and neuron LPS-treated groups that maintained a high level of inclusion were from the same animal, ‘LPS6'. (**c**) −Δ*C*_t_ qPCR results for example event in *Sltm*. Constitutive (C), skipping (S) and inclusion (I) assays were analysed in a replication cohort of four animals per treatment. Lines connect results for individual animals. Greater −Δ*C*_t_ values indicate higher abundance of the corresponding *Sltm* isoform. Higher ‘S' values, relative to ‘I' values, in LPS samples indicate more skipping. (**d**) ΔΔ*C*_t_ qPCR results, defined as (−Δ*C*_t_^inclusion^)−(−Δ*C*_t_^skip^), for same *Sltm* event confirm increased exon skipping upon LPS treatment. Lower ΔΔ*C*_t_ values indicate more skipping, as seen in the LPS-treated samples. (**e**) Differential splicing events identified by RNA-Seq and tested by RT–qPCR. Variant frequency (vFreq) and ΔΔ*C*_t_ for each event were Z-score normalized within each cell type. White colour indicates average inclusion for that event and cell type; red or blue indicate above average inclusion or skipping, respectively. (**f**) Summary of qPCR results for validating the LPS-induced RNA splicing alterations identified by RNA-Seq. qPCR results are summarized based on average ΔΔ*C*_t_ for direction of change and significant (sig.) or not based on adjusted *t*-test *P*≤0.05. Cont., control.

Surprisingly, we identified significant changes for hundreds of known and novel events in all three cell types (Supplementary Data 3). In contrast with gene expression changes, neurons showed as many RNA processing changes as the glial cell types. Many of these changes were in common between the cell types, suggesting a partially shared RNA processing response to peripheral endotoxemia among CNS cell types. For example, we identified a differentially spliced cassette exon in the *Sltm* gene (Fig. 7a), which encodes a protein suggested to play a role in global transcriptional regulation as well as apoptosis^27^. In all three cell types from vehicle-treated animals, nearly all of the splice junction reads observed near this exon aligned to the inclusion isoform, whereas in LPS-treated animals many reads aligned to the skipping isoform (Fig. 7a). The extent of skipping in LPS-treated animals was variable and correlated with the overall transcriptional response as the LPS6 animal, which exhibited the weakest LPS response by gene expression (Supplementary Fig. 6), showed no skipping of this exon in either microglia or neurons (Fig. 7b).

To validate these findings, we selected 27 LPS-induced splicing events that were significant in one or more cell types and for which we could design qPCR assays (see the Methods for details), including the *Sltm* event, and we analysed a new cohort of vehicle- and LPS-treated animals. For each event, we designed assays targeting the flanking constitutive region (C) as well as the skipping (S) and inclusion (I) isoforms. Using this new cohort and different technology, the overall pattern of LPS-induced splicing for these 27 events was validated in all three cell types (Fig. 7c–f; see also Supplementary Data 4 for details). Nearly all events identified as differentially spliced by RNA-Seq showed the same trend by RT–qPCR in microglia (13 of 13 events), neurons (17 of 17 events) and astrocytes (16 of 18 events), with 85, 76 and 17% of the events reaching significance in their respective cell types by RT–qPCR (adjusted *P*≤0.05). We excluded the possibility that our findings were an artefact of our dissociation or fixation methods by confirming that most of the events were LPS-induced even in whole cortex RNA extracted directly from intact, opposite hemisphere of the same brains (see Supplementary Data 4 for details). Thus, peripheral endotoxemia produces a robust and specific RNA processing response in the CNS.

### TNFR1 required for astrocytic response to endotoxemia

**Figure 8:**
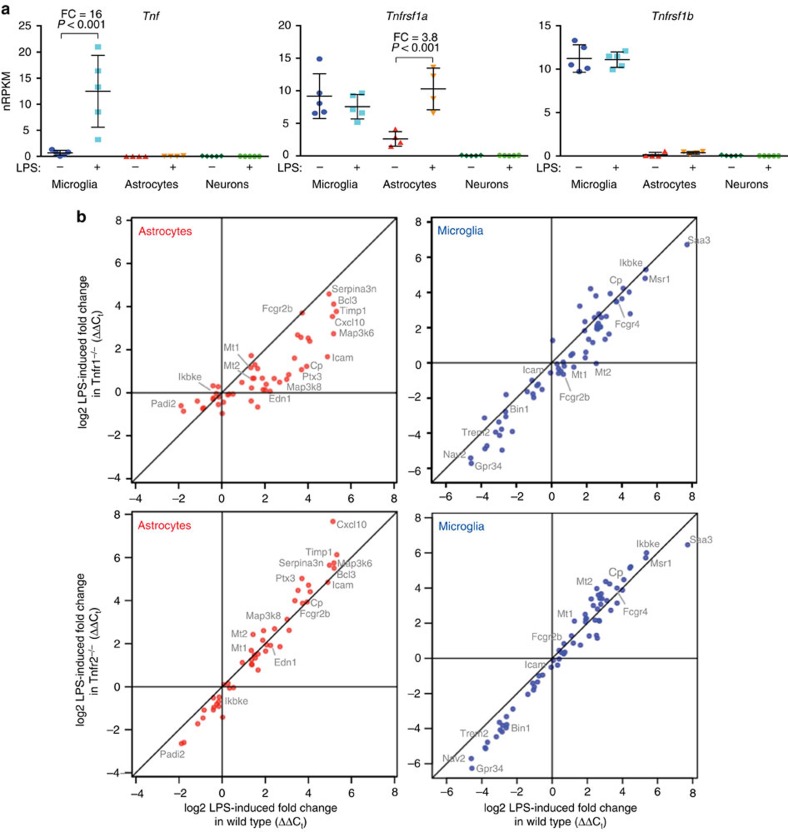
TNF receptor and cell type specificity in CNS endotoxic response. (**a**) RNA-Seq data showing expression levels of TNF, Tnfr1 and Tnfr2 in microglia and astrocytes, with LPS-induced fold changes (FCs) and adjusted *P*-values shown (Wald test, DESeq2; *n*=5 animals per treatment). Neuronal expression of Tnf receptors was negligible. Bars represent mean±s.d. (Prism). (**b**) Wild-type and TNFR knockout colony mates were injected with saline or LPS (*n*=5 per treatment and genotype). Cell types were purified 1 day post injection and tested by RT–qPCR for transcriptional responses, normalized against averaged *C*_t_ values for *Gapdh* and *Rpl37a*. Comparing LPS-induced changes (ΔΔ*C*_t_=average−Δ*C*_t_^LPS^ minus average −Δ*C*_t_^saline^) between wild-type and knockout cell types revealed a strong dependence on Tnfr1 for the much of the astrocytic response (top left plot). Tnfr1^−/−^ microglia responded normally overall (top right plot), although a few genes (see *Mt2*) displayed Tnfr1 dependence. The absence of Tnfr2 had no strong effect on any cell type (bottom plots).

To demonstrate this method's utility for dissecting CNS inflammatory signalling mechanisms, we explored whether the TNF pathway is required for the brain's endotoxic response in each cell type. *Tnf* was expressed at low levels in control microglia and escalated in response to endotoxemia (Fig. 8a). As for the receptors, *Tnfrsf1a* (Tnfr1) was expressed most prominently in microglia and at a lower level in astrocytes, which increased during endotoxemia to a level approximating that of microglia. *Tnfrsf1b* (Tnfr2) expression was highly enriched only in microglia. Notably, neuronal expression of Tnfr1 and Tnfr2 was negligible, suggesting that reported neurotoxic and neuroprotective activities of Tnfr1 and Tnfr2, respectively^28^,^29^, likely occur through indirect signalling mechanisms.

The induction of *Tnf* in microglia led us to test whether TNF receptors contributed to the brain's endotoxic transcriptional response. We injected LPS into wild type, *Tnfrsf1a*^*−/−*^, *Tnfrsf1b*^*−/−*^ or *Tnfrsf1a*^*−/−*^*;Tnfrsf1b*^*−/−*^ mice (colony mates); FACS-purified cortical cell types 24 h post injection; and analysed expression by RT–qPCR for a set of genes with a variety of LPS responses in different cell types. The microglial response was largely Tnfr-independent, as only a few of the genes we analysed showed impaired responses in microglia lacking Tnfr1 or Tnfr2 (Fig. 8b). In contrast, astrocytes from mice lacking Tnfr1 were markedly impaired in their transcriptional response to endotoxemia, whereas this response was unimpaired in mice lacking only Tnfr2 (Fig. 8b). It is tempting to speculate that the Tnfr1-dependent astrocytic response resulted from direct TNF/Tnfr1 signalling in astrocytes, which express Tnfr1 but not Tnfr2. However, some of the dependence on Tnfr1 may be indirect since neurons, in which Tnfr1 expression is negligible, also displayed certain LPS-induced changes that were Tnfr1-dependent (data not shown). Data from the Tnfr1/Tnfr2 double knockout mice were highly similar to the Tnfr1 knockout data for all cell types. In summary, the brain's transcriptional response to endotoxemia showed a strong requirement for Tnfr1 in the case of astrocytes but not microglia and was Tnfr2-independent in all cell types tested.

### Differential expression by cell type in Alzheimer's model

**Figure 9:**
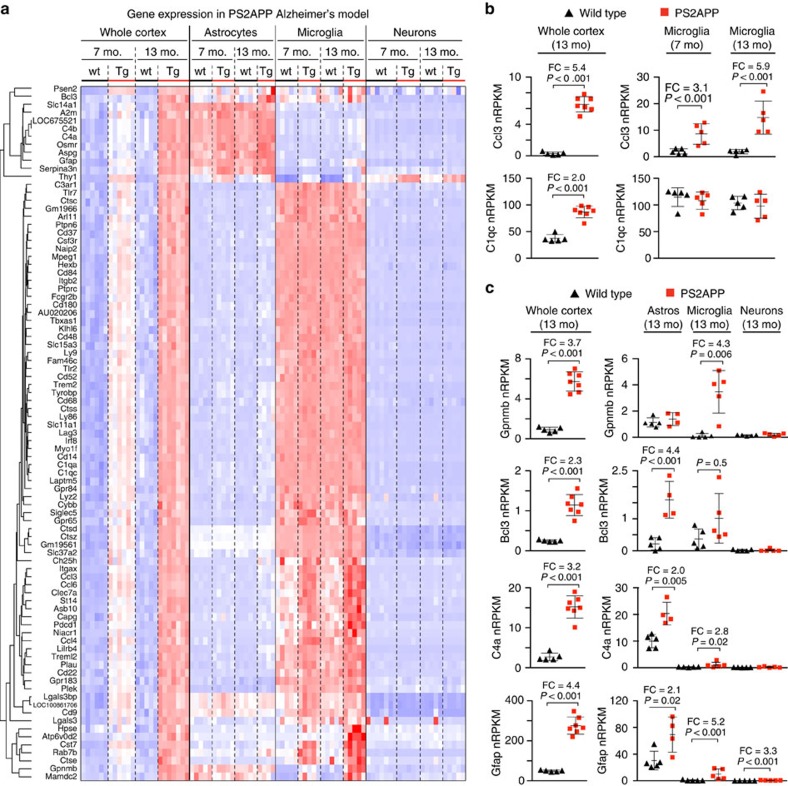
Gliosis entirely accounts for amyloid-driven changes in whole tissue RNA profiles. (**a**) Heat map of RNA-Seq data relating the genes with ⩾2-fold change (FC; adjusted *P*≤0.05) in 13-month-old PS2APP whole cortex (GSE75357, this study) to expression data for astrocytes, microglia and neurons acutely isolated from aged transgenic (Tg) and wild-type (wt) mice (GSE75431, this study). Most genes with increased abundance in PS2APP cortex were preferentially expressed by microglia, but only a fraction of these were transcriptionally upregulated in microglia. Expression was *Z*-score normalized for each gene, separately within each of the two data sets (colour range: −2.03≤Z≤4.00). One astrocyte sample from PS2APP mice was excluded from the analysis due to evidence of cell type contamination. (**b**,**c**) Expression patterns of indicated genes analysed by RNA-Seq for whole cortex and for sorted cells, from PS2APP versus non-transgenic cortices, with FC and adjusted *P*-values (Wald test, DESeq2; *n*=5 animals per genotype) shown and bars representing mean±s.d. (Prism). Note: Absolute nRPKM values between whole cortex and sorted cell studies should not be directly compared, due to different library preparation methods.

Finally, we explored the utility of this method in the context of neurodegenerative disease by obtaining RNA-Seq data from both bulk tissue and FACS-purified cell types from 7- and 13-month-old mouse brains in the PS2APP model of AD (Fig. 9a). In 13-month-old PS2APP whole cortex, we detected 85 genes (in addition to the transgene elements) with ⩾2-fold increased RNA abundance (adjusted *P*≤0.05)—a larger number than the 51 genes detected by microarray (Fig. 1c). Of these 85 genes, 66 showed specific or highly enriched expression in microglia compared with astrocytes and neurons sorted from either non-transgenic or PS2APP cortex of that age. However, only a fraction of these genes (13 out of 66) showed increased expression (adjusted *P*≤0.05) in PS2APP microglia (for example, *Ccl3*, *Clec7a*, *Treml2*), whereas the remainder showed no clear changes (for example, *C1qc*, *Cybb*, *Gpr84*, *Ptprc*, *Trem2*), with 20 out of 53 trending downward and 33 out of 53 trending upward (see Fig. 9b for examples). These data directly support our earlier conclusions that the changes in RNA profiles observed in whole tissue samples for neurodegenerative diseases are driven more by relative cell type abundance than by disease-related transcriptional modulation.

Of the remaining 19 genes with ⩾2-fold higher detection in PS2APP whole cortex, microglia were responsible for 8 of them, including *Gpnmb* and *Mamdc2*, which normally show astrocyte-enriched expression (see *Gpnmb* in Fig. 9c). In all, microglia accounted for 87% (74 out of 85) of the changes observed in whole tissue, because of increased microglial cell number, increased expression levels within microglia or both. Astrocytes accounted for most (8 out of 11) of the remaining changes—roughly 10% of total—with 5 genes including *Gfap*, *Bcl3* and complement 4 reaching significance (Fig. 9c) and 3 trending strongly upward in sorted PS2APP astrocytes (not shown). Three changes in whole tissue could not be conclusively assigned to any cell type, but all three were expressed selectively by glia. Of note, neurons accounted for none of the expression changes observed in PS2APP tissue except for that of the transgene. (PS2APP neurons as well as microglia showed elevated *Gfap* expression, with fold changes higher than in astrocytes, but the increased *Gfap* signal in whole tissue was assigned primarily to astrocytes due to their higher absolute expression level.)

**Figure 10:**
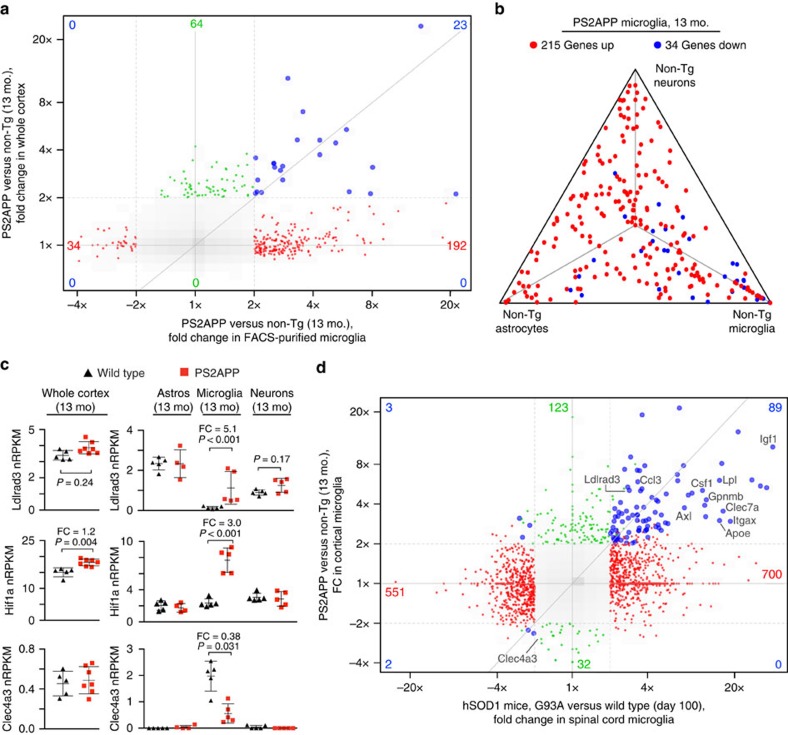
Analysis of amyloid-driven changes in microglial gene expression. (**a**) Genome-wide ‘four-way' plot comparing differential gene expression (fold change (FC) ⩾2, adjusted *P*≤0.5) observed in PS2APP whole cortex versus that observed in purified PS2APP microglia (each compared with non-transgenic). Each point represents a single gene, with only genes meeting the cutoffs in either study being plotted. Red, green and blue points met the cutoffs in sorted microglia only, whole cortex only or in both analyses, respectively. Grey indicates the distribution of FC values for all genes. (**b**) In this triangle plot, the 249 genes differentially expressed in PS2APP microglia (FC ⩾2, adjusted *P*≤0.05) are plotted to show their degree of preferential expression in neurons, astrocytes or microglia from normal, non-transgenic (non-Tg) cortex. (**c**) Specific examples of amyloid-driven changes in microglial gene expression that were undetected or muted in whole tissue expression profiles, with FC and adjusted *P*-values (Wald test, DESeq2; *n*=5 animals per genotype) shown and bars representing mean±s.d. (Prism). Note: Absolute nRPKM values between whole cortex and sorted cell studies should not be directly compared, because of different library preparation methods. (**d**) Similar to **a**, except this ‘four-way' plot compares genes differentially expressed in microglia from PS2APP Alzheimer's model (this study) with those observed in microglia from the hSOD1^mut^ ALS model (GSE43366, imported).

More interestingly, in sorted PS2APP microglia we observed more than 200 genes with ⩾2-fold change in expression (adjusted *P*≤0.05) that were undetected or less apparent in whole tissue (Fig. 10a). Many of these were constitutively expressed in neurons and/or astrocytes, explaining why their upregulation in microglia was difficult to observe in whole cortex (Fig. 10b). Increased expression of *Apoe*, *Lpl* and *Ldlrad3* in PS2APP microglia (example in Fig. 10c) suggested that microglia from plaque-ridden cortical tissue have altered lipoprotein metabolism and may contribute to pathogenic amyloid precursor protein (APP) processing and amyloid deposition^30^,^31^,^32^,^33^. PS2APP microglia also upregulated the genes encoding Igf1, the transcription factor Hif1a and its partner Arnt2 (ref. 34), and the transcriptional repressor Bhlhe40 and its partner Arntl (ref. 35; example in Fig. 10c). Finally, among the genes with altered expression in PS2APP microglia were 34 instances of genes with ⩾2-fold decreased expression. These included microglia-specific transcripts such as *Clec4a3*, *Irf4*, *Mgl2*, *Mrc1* and *Tgm2*, none of which showed decreased abundance in whole tissue RNA as the decreases in expression were offset by increased microglial cell number in PS2APP cortex (example in Fig. 10c). These examples clearly illustrate the advantageous view of neurodegenerative disease that cell type-specific expression profiles can provide, which cannot be appreciated using whole tissue expression profiles.

Looking at microglial gene expression more broadly, we compared the pattern of differential expression in purified PS2APP cortical microglia with that of microglia purified from spinal cords of hSOD1^mut^ mice^23^ to look for evidence of similar gene regulation. Despite the unique aetiologies and locations of the two pathologies, and the fact that little to no neuronal loss occurs in PS2APP brains, we observed significant overlap between the ‘neurodegeneration signature' described by Chiu *et al*. and the differential expression pattern in PS2APP microglia. Over 40% of the upregulated genes (⩾2-fold, *P*≤0.05) in PS2APP microglia were also upregulated in hSOD1^mut^ microglia (Fig. 10d). Conversely, only 11% of the genes upregulated in hSOD1^mut^ microglia were also upregulated in PS2APP microglia, presumably reflecting the more aggressive pathological and neurodegenerative phenotypes in the ALS model compared with the AD model.

## Discussion

Studying whole tissue transcriptomes from neurodegenerative disease specimens has frequently led to the conclusion that inflammation-related pathways are active in the disease. However, whether these changes are causal or consequential in the disease process has not been clear. Our examination of existing data sets from neurodegenerative disease tissues revealed a common trend—that genes with decreased mRNA abundance were typically expressed by neurons, and genes with increased mRNA abundance were typically expressed by microglia or astrocytes—suggesting that many changes resulted not from transcriptional modulation but from neuronal loss and the attendant increase in glial cell fraction. Supporting this interpretation, we observed bulk enrichment of microglia- and astrocyte-specific transcripts and depletion of neuron-specific transcripts in these tissue profiles. We also revealed potential limitations of laser-capture microdissection, as most of the transcripts with increased abundance in ALS motor neuron samples likely originated from gliotic cell fragments that were unintentionally co-excised with the neurons.

To illuminate the brain's cellular mechanisms of inflammatory signalling, we improved on a method to dissociate and immunopurify CNS cell types from adult mouse brain, followed by RNA purification, amplification and sequencing. The method avoids unwanted changes to the transcriptome that occur during enzymatic dissociation at warm temperatures, and it does not require genetic labelling of any cell type. Fixation after dissociation enables the labelling of intracellular targets and prevents additional changes to RNA that may occur during labelling and sorting. No pooling of samples is required, so treatment responses can be correlated between cell types within individual animals and compared between animals. Using peripheral endotoxemia as a model to prove this method, we demonstrated that the brain's responses in gene expression and RNA processing are exquisitely cell type specific, and we observed myriad changes that would go undetected without cell sorting.

A caveat of the method is whether the population of cells recovered after dissociation and sorting adequately represents the starting population. The number of cells that survive the harsh dissociation procedure is a fraction of the cells originally present; it seems likely that subpopulations within each cell type will have different liabilities. Moreover, only cell body-contained transcripts will be recovered, as cellular extensions such as axons and dendrites are sheared during mechanical dissociation. Another consideration is immunolabelling specificity—NeuN is expressed in most, but not all, neurons; CD11b is also expressed by peripheral myeloid cells that may infiltrate the brain; some astrocytes express little GFAP under normal conditions. We recovered similar numbers of GFAP^+^ cells from saline and LPS-injected animals, arguing against the notion that LPS-induced changes in astrocyte gene expression resulted from additional subtypes being collected after GFAP upregulation (data not shown). Numbers of CD11b^+^ cells recovered after LPS injection were also similar, although a small percentage was CD45^high^, suggesting a slight contribution from peripheral myeloid cells (data not shown).

The mechanisms by which peripheral endotoxin induces a CNS transcriptional response are imprecisely understood. A pervasive misconception is that astrocytes can directly respond to LPS exposure^36^,^37^,^38^, but recent data have clarified that microglia are critical intermediaries in the astrocytic response^39^; our expression data confirm that astrocytic expression of the LPS co-receptors Tlr4 and CD14 is extremely low (not shown). The intermediary signals between stimulation of immune cells by LPS and the endotoxic astrocyte response are unknown, but using our method we established a requirement for Tnfr1 in the indirect process of LPS-induced astrocyte activation. The microglial response was independent of TNF receptors, consistent with the possibility of direct exposure to LPS following LPS-triggered permeabilization of the blood–brain barrier^40^.

Extending the method to a murine AD model, we verified that the gene expression changes observed in whole cortex RNA resulted mostly from increased numbers of microglia in PS2APP cortex, sometimes in combination with increased expression within microglia. This was yet another indication that many of the ‘hits' identified in whole tissue expression data are trivial, unavoidable consequences of altered cell type composition. Importantly, the vast majority of transcripts with altered expression in sorted PS2APP microglia were not significantly altered in whole tissue RNA, confirming the importance of obtaining cell type-specific expression data for revealing disease-related transcriptional changes. Comparing our data with published data from an ALS mouse model revealed that many of the microglial expression changes were conserved between models of ALS and AD, suggesting a common microglial response to distinct disease pathologies.

The approach we have described will allow researchers to identify bona fide instances of disease-related gene regulation, pinpoint the cell types in which those change occur and formulate better hypotheses for the cellular mechanisms of neurodegenerative disease. Applying this method to additional models and patient tissues will potentially transform our understanding of the role of aberrant gene expression in neurological disease or injury.

## Methods

### Mice

All protocols involving animals were approved by Genentech's Institutional Animal Care and Use Committee, in accordance with guidelines that adhere to and exceed state and national ethical regulations for animal care and use in research. Eight- to ten-week-old C57BL/6 males were injected intraperitoneally with LPS (0111:B4 L3024, Sigma) at 10 mg kg^−1^. Control animals were injected with an equal volume of PBS. Animals were monitored for 24 h and perfused with PBS before dissection. Animals were perfused and processed in pairs of one LPS- and one PBS-injected mouse at a time, and sorted in two BD FACSAria sorters simultaneously. TNF receptor knockout mice were obtained from Jackson (stock no. 003243) and maintained as a colony to ensure production of both knockout and wild-type mice. For cell sorting studies using PS2APP^14^ model, female cohorts (except one male in 13-mo non-transgenic group and two males in 13-mo transgenic group) were perfused and processed at 7 and 13 months, usually in pairs of same-age transgenic and non-transgenic mice. For microarray studies using PS2APP model, male cohorts were perfused and processed for whole hemicortex RNA purification at 3, 7 and 13 months. For bulk tissue RNA-Seq studies using PS2APP, female cohorts were similarly processed at 7 and 13 months. In general, using 4–6 mice per treatment group or genotype was enough animals to detect robust changes in gene expression as statistically different.

### Preparing dissociated cell suspensions from adult mouse brain

Immediately following perfusion with PBS, cortical caps (including hippocampi) were rapidly dissected on ice, minced into smaller pieces using a fresh chilled razor blade, and transferred to a round-bottom 2 ml Eppendorf tube containing 1.6 ml of Accutase (SCR005, Millipore). The tissue was rotated at 4 °C for 20 min and spun at 2,000 relative centrifugal force (r.c.f) for 1 min in a refrigerated centrifuge. The supernatant was discarded and 1.5 ml Hibernate A Low Fluorescence medium (BrainBits) was added to the tissue. The tissue was manually and carefully triturated using first a blunted 1 ml tip five times followed by an uncut 1 ml tip seven or eight times. The cell suspension was kept on ice for 10–15 s to allow the larger tissue to settle. The top cloudy 1 ml cell suspension was removed and passed through a 70-μm pre-wetted filter. A fresh 1 ml of Hibernate A was then added to the cell suspension and the trituration was repeated until a total of 4 ml of cell suspension was obtained. This cell suspension was pipetted onto a discontinuous Percoll gradient (described in Guez-Barber, *et al*.^12^) in a 15-ml Falcon tube, high density on bottom, and centrifuged at 430 r.c.f. for 4 min in a tabletop centrifuge, followed by removal and discarding of the top 2 ml of cloudy liquid. The remaining 5 ml was centrifuged at 550 r.c.f. for 4 min. The resulting supernatant was discarded and the cell pellet was resuspended gently in 1 ml of ice cold Hibernate A. One millilitre of ice-cold 100% ethanol was added drop by drop while gently swirling the falcon tube containing cells on ice to uniformly and gently fix the cells. After the cells were mixed to a final concentration of 50% ethanol, the falcon tube was kept on ice for 15 min. The fixed cells were centrifuged at 550 r.c.f. for 5 min, and the resulting supernatant was discarded. The cell pellet was resuspended gently in 10 ml of ice-cold Hibernate A to wash residual ethanol, centrifugation was repeated and cells were finally resuspended in 1 ml of ice-cold Hibernate A and aliquoted for immunostaining (50 μl cells+950 μl Hibernate A for isotype and unstained control cells; remaining 900 μl of cells+100 μl of Hibernate A for antibody staining).

### Dissociated cell immunostaining, FACS and RNA isolation

The following antibodies were used for immmunostaining neurons, astroctyes and microglia, respectively: AlexaFlour 488-conjugated anti-NeuN (MAB377X, Millipore) at 1:1,000, PE-conjugated anti-GFAP (561483, BD Biosciences) at 1:50 and APC-conjugated anti-CD11b (561690, BD Biosciences) at 1:250. 1 μl of DAPI (1 mg ml^−1^ stock) was added to all the tubes. The cells were rotated at 4 °C for 20 min. Cells were centrifuged at 2,000 r.c.f. for 2 min in a refrigerated centrifuge followed by two brief washes in 1 ml of Hibernate A. Cells were resuspended in 3 ml of Hibernate A and passed through a 40-μm filter before sorting. Cells were sorted using DAPI^+^ signal as a gate to select for singlet cell bodies and ignore doublets and cell debris. Using this group as the parent gate, further FACS gates were determined relative to isotype controls. CD11b^+^ cells were distinct from other cell types. CD11b^–^ cells separated into distinct NeuN^+^ and NeuN^–^ clusters, and neurons were gated from the NeuN^+^ cluster. Astrocytes were gated from the GFAP^high^ cells that were also NeuN-negative. All cell types were finally gated against other fluorescent gates to get the final population. In 13 months PS2APP versus non-transgenic mice, the CD11b^+^ population included cells with increased GFAP^+^ signal (although still lower than astrocytic GFAP^+^ signal) but these were gated out to ensure that collected microglia did not include contaminating astrocytic material. Cells were sorted into protein LoBind tubes (022431081, Eppendorf) containing 50 μl of Hibernate A medium. Cells were centrifuged at 2,600 r.c.f. for 7 min (neurons) or 6,600 r.c.f. for 7 min (other cell types) and the supernatant was discarded. RNA was extracted from the cell pellet using Qiagen RNeasy Plus Micro kit in a final volume of 25 μl. We typically obtained 200–300 ng RNA from neurons, 10–20 ng from microglia and 2–5 ng from astrocytes. RNA quality was quantified using the Bioanalyzer.

### Reverse transcription and pre-amplification

RNA (typically, 7.5 μl in a 20 μl total reaction volume) from sorted cells was reverse transcribed using the High-Capacity cDNA Reverse Transcription kit (4368814, Applied Biosystems). Standard Taqman FAM-MGB probes were ordered from Applied Biosystems for all qPCR assays, except our assays to analyse LPS-induced alternative splicing were designed and synthesized at Genentech using FAM-BHQ1 for probe detection. For pre-amplification, up to 100 qPCR assays (primer/probe sets in 20 × stock concentration) were pooled and diluted to a 0.2 × concentration. For splicing analyses, separate assay pools were constructed for constitutive, inclusion and skipping isoforms so that different isoforms from the same gene were never in direct competition for primer/probe interaction during pre-amplification. Five microlitres of pooled assays were combined with 2.5 μl of sample cDNA, 10 μl of 2 × TaqMan PreAmp Master Mix (1410056, Applied Biosystems) and 2.5 μl of TE buffer, and pre-amplified for 14 cycles using the cycling conditions recommended by Applied Biosystems, in a 96-well plate (N8010560, Applied Biosystems) sealed tightly with MicroAmp Clear Adhesive films (4306311, Applied Biosystems). No cDNA pre-amplification was performed for qPCR reactions derived from whole tissue RNA samples.

### Fluidigm qPCR analyses

Fluidigm reactions were performed using the 96 × 96 or 48 × 48 chips and included 2–3 technical replicates for each combination of sample and assay, except splicing analyses had 6 technical replicates. For sample mixtures, 2.5 μl pre-amplification product from sorted cell samples or 1.5 μl undiluted reverse transcription product from whole tissue samples, was combined with 20 × GE Sample Loading Reagent (85000735, Fluidigm), 2 × PCR master mix (58003365-01, Applied Biosystems) and TE buffer in a 10-μl volume, of which 5 μl was loaded into sample wells. For assay mixtures, equal volumes of TaqMan assay (or custom assay for splicing analyses) and 2 × Assay Loading Reagent (85000736, Fluidigm) were combined, and 5 μl of the resulting mixture was loaded into multiple assay wells for technical replicates. Data from Fluidigm runs were manually checked for reaction quality before analysis, and *C*_t_ values for each gene target were normalized to *C*_t_ values for housekeeping genes. Data analysis for gene-level expression was performed using Microsoft Excel software and plotted using GraphPad Prism 6 software. Data analysis for splicing detection assays is described below.

### RNA library prep and sequencing

Total RNA extracted from sorted astrocytes, microglial and neuronal cells was subjected to RNA-Seq. The concentration of RNA samples was determined by NanoDrop 8,000 (Thermo Scientific). The integrity of RNA samples was tested using Bioanalyzer RNA 6,000 Pico Kit (Agilent). RNA Integrity Number (RIN) varied between the samples from 3 to 8.5 where RNA from neurons showed lower quality (average RIN ∼4) compared with RNA from astrocytes (average RIN ∼5.5) and microglia (average RIN ∼6.5). cDNA was generated from up to 25 ng of total RNA using Nugen's RNA-Seq method for low-input RNA samples, Ovation RNA-Seq System V2 (NuGEN). (Per manufacturer's instructions, total RNA was neither depleted of rRNA nor polyA-selected.) Generated cDNA was sheared to 150–200 bp size using LE220 focused ultrasonicator (Covaris). Following cDNA shearing, the size of samples was determined using Bioanalyzer DNA 1,000 Kit (Agilent). In addition, sheared cDNA samples were quantified by Qubit dsDNA BR Assay (Life Technologies). One microgram of sheared cDNA was taken into further processing, starting at end repair step, using Illumina's TruSeq RNA Sample Preparation Kit v2 (Illumina). Generated libraries were amplified using six cycles of PCR. Size of the libraries was confirmed using Tapestation (Agilent).

For sequencing of bulk tissue RNA, total RNA was extracted from intact cerebrocortical tissues of PS2APP and non-transgenic mice at 7 and 13 months age. Concentration of RNA samples was determined using NanoDrop 8,000 (Thermo Scientific) and RNA integrity was determined by Fragment Analyzer (Advanced Analytical Technologies). 0.5 μg of total RNA was used as an input material for library preparation using TruSeq RNA Sample Preparation Kit v2 (Illumina). Size of the libraries was confirmed using Fragment Analyzer (Advanced Analytical Technologies).

Library concentrations were determined by qPCR-based method using Library quantification kit (KAPA). The libraries were multiplexed and then sequenced on Illumina HiSeq2500 (Illumina) to generate 30 M of single end 50 base pair reads per library.

### RNA-Seq alignment and feature counting

HTSeqGenie^41^ was used to perform filtering, alignment and feature counting. HTSeqGenie uses GSNAP^42^ to align reads to the genome. We used version mm9 of the mouse genome, and gene models from our internal database, mostly based on RefSeq. The coordinates of these models are available in Supplementary Data 5 (mouse). Only reads with unique genomic alignments were analysed. Such reads whose alignments overlapped any exon of the gene model (even by a single base) were counted towards that gene. (For comparison, we also ran our pipeline with mm10, and the results were found to be similar.) In the LPS study (GSE75246), two astrocyte samples—one from saline and one from LPS-injected—were excluded from the analysis because of evidence of cell type contamination. In the PS2APP study (GSE75431), one astrocyte sample from the PS2APP group was excluded from the analysis for the same reason.

### RNA-Seq normalization

nRPKM (normalized Reads Per Kilobase gene model per Million total reads) values were used as a normalized measure of gene expression (except for heat maps, see below). This statistic is an attempt to combine the best of DESeq sizeFactor^43^-normalized counts and traditional RPKM. Whereas RPKM is defined as


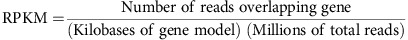


(where ‘total' means ‘total uniquely aligned'), the *normalized* RPKM is defined as


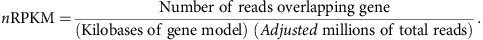


Note that the only difference is in the ‘*M*' term. The adjusted total reads is a statistic which is proportional to DESeq sizeFactor, but rescaled to the level of total uniquely aligned reads:


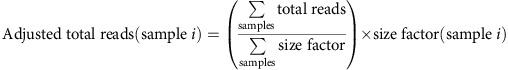


Note that this is a single normalization based on size factors, with an overall adjustment to approximate total reads, but is not a double normalization.

For projects where the size factor is highly correlated with total reads, nRPKM will be very close to the RPKM. For projects where there is a discrepancy, typically due to fluctuations in a few very highly expressed genes, the two statistics will be different. In either case, the nRPKM values for one gene are exactly proportional to the ‘normalized count' statistic used by some other authors, which is just number of reads overlapping a gene divided by the size factor. Furthermore, we remind the reader that care should be taken in comparing nRPKM values between projects (that is, between groups of samples which were not normalized together). In particular, our amplification method results in large numbers of intronic reads, so the *M* value is relatively higher (and nRPKM values relatively lower) than a typical polyA-selected RNA-Seq library.

For cufflinks/cuffdiff users, our ‘traditional' RPKM statistic is analogous to the classic-fpkm statistic generated by cufflinks, whereas the nRPKM statistic is analogous to the FPKM statistic provided by the cuffdiff package with geometric normalization (see http://cole-trapnell-lab.github.io/cufflinks/cuffdiff/#library-normalization-methods for details). For an in-depth comparison of these methods, see Supplementary Data 6. For DESeq2 users, our nRPKM gives a matrix which is the same as the one returned by the fpkm function, multiplied by a constant which is the ratio of total uniquely alignable reads (which we use to calibrate the *M*) to the sum of all gene counts (which DESeq2 uses to calibrate the *M*).

### RNA-Seq differential gene expression

Differential gene expression was performed with DESeq2 (ref. 43). A pre-filter was applied: only genes with at least ten counts in at least three samples (of either condition) were analysed. *P*-values for other genes were simply set to 1 and log-fold-changes to 0 for visualization purposes, but such genes were not included in the multiple testing correction.

### RNA-Seq splice isoform prediction

Splicing events were predicted and quantified by SGSeq^25^. SGSeq yields ‘splicing events' (or just ‘events'), which are composed of multiple (but usually just 2) variants. For example, a classical cassette alternative exon would be considered a single event, and the inclusion and skipping isoforms considered the ‘splice variants' (or just ‘variants'). The variants in turn are composed of ‘splice graph features' (or just ‘features'). For visualization purposes (for example, see Fig. 7), variants were quantified with the variantFreq statistic, which ranges from 0 (fully skipped) to 1 (fully included). variantFreq was summarized with R's box-plot function: boxes show median and interquartile range, and whiskers show full data.

### RNA-Seq differential splicing analysis

To identify differential splicing events, first a single count for each variant in each sample was determined by adding together the 5′ and 3′ counts for each variant, or, if they represented the same features, by simply taking the single unique value. These counts were then analysed using a variation on the DEXSeq package^26^. Normally (for example, looking at the Bioconductor vignette or manuscript), DEXSeq analyses differential usage of *exons* across a single *gene.* In our case, we analysed differential usage of *variants* across a single *event*. In fact, within the DEXSeq vignette and manuscript one can find suggestions of using the tool in such a manner, so although not typical, this is not a new idea. As with the differential gene expression, a pre-filter was applied: only variants with at least five counts in at least three samples (of any condition) were analysed. Then, any events for which only a single variant remained were considered to be effectively constitutive and that remaining variant was discarded. For most comparisons these two filters greatly reduced the total number of variants tested, to around 6,000. Then, DEXSeq was run on the remaining data using the SGSeq event as the DEXSeq ‘gene' and the SGSeq variant as the DEXSeq ‘exon'. The model used was sample+exon+condition : exon. After running DEXSeq, to limit the number of tests, the first variant of each event (generally a ‘skipping' variant) was discarded. As our data had high levels of intronic reads, possibly due to the Nugen amplification, retained intron events were also discarded.

### Fluidigm splicing assay primer design

For each target event, primers were designed using the Primer3 programme^44^ (Supplementary Table 1). ‘Constitutive' assays targeted constitutively spliced regions within 300 bases of, but not overlapping, the alternative event. For ‘Inclusion' assays, one primer was designed to anneal to the inclusion region, the other to the flanking constitutive region, and the detection oligo anywhere between (not necessary overlapping a splice junction). For ‘Skipping assays', one primer was designed to anneal to the splice junction, with the default required overlaps (that is, at least 4 bases on the 3′ end and at least 7 on the 5′ end). In addition, the following non-default physical parameters were provided to primer 3:

PRIMER_INTERNAL_MIN_TM=68

PRIMER_INTERNAL_OPT_TM=70

PRIMER_INTERNAL_MAX_TM=72

PRIMER_INTERNAL_MIN_SIZE=24

PRIMER_INTERNAL_OPT_SIZE=28

PRIMER_INTERNAL_MAX_SIZE=36

PRIMER_INTERNAL_MUST_MATCH_FIVE_PRIME=hnnnn

PRIMER_PRODUCT_SIZE_RANGE=62–85 86–100 101–120 120–140

### qPCR splicing data analysis

Amplification curves were visually inspected and pass/fail calls were adjusted as appropriate. Technical replicates were averaged together, omitting fail calls, to get basic *C*_t_ values for each assay/sample pair. Assays that failed on all technical replicates for a sample were imputed at *C*_t_^max^+1, where *C*_t_^max^ was the highest *C*_t_ (passing) value achieved for that assay in any sample. These imputed values are indicated with different symbols in the plots. −Δ*C*_t_ values were generated by subtracting the average *C*_t_ values for the *Actb* loading control and taking the inverse, and ΔΔ*C*_t_ values were calculated as (−Δ*C*_t_^inclusion^)−(−Δ*C*_t_^skip^). Imputed −Δ*C*_t_ and ΔΔ*C*_t_ values are indicated with different symbols in the plots. Student's *t*-test was performed for each cell type comparing the ΔΔ*C*_t_ values from vehicle and LPS-treated samples (including imputed values), and *P*-values were adjusted for multiple testing using the Benjamini–Hochberg method. In Fig. 7, these statistics were summarized with R's boxplot function: boxes show median and interquartile range, and whiskers show full data range (generated using R's boxplot function).

### Whole tissue RNA purifications

For comparison of LPS response in whole cortex versus sorted cell populations, fragments of cortical tissue were weighed, minced and added to appropriate volume of lysis buffer RLT, and 350 μl of lysate was used for purification according to the RNeasy Micro Plus kit and protocol (Qiagen). For comparison of PS2APP transgenic versus non-transgenic RNA from intact cortical tissue, hemicortices were rapidly frozen on dry ice and stored until a full collection was obtained. RNA was extracted using the RNeasy Lipid Tissue extraction kit with on-column DNase digestion (Qiagen). Quality and quantity of total RNA samples were determined using ND-1000 spectrophotometer (Thermo Scientific) and Bioanalyzer 2100 (Agilent), respectively.

### Microarray RNA processing, hybridizations and scanning

The method for preparation of Cy-dye-labelled cRNA and array hybridization was provided by Agilent. Briefly, 1 μg total RNA was converted to double-stranded cDNA and then to Cy5-labelled cRNA using Quick Amp Labeling Kit (Agilent). The labelled cRNA was purified using RNeasy mini kit (Qiagen). cRNA yield and Cy5 incorporation were determined using ND-1000 spectrophotometer. 750 ng of labelled cRNA was fragmented and hybridized to Agilent's Whole Mouse Genome 4 × 44Kv2 arrays as described in the manufacturer's hybridization kit, against an equal amount of Cy3-labelled universal mouse reference (Stratagene). Following hybridization, microarrays were washed, dried and scanned on Agilent's G2505C scanner. Agilent's Feature Extraction 11.5 software was used to analyse acquired array images.

### Microarray analysis

For Affymetrix data, CEL files were downloaded from Gene Expression Omnibus (GEO) and normalized using the rma algorithm^45^. Probeset-gene mappings were derived from bioconductor. For Agilent data, TXT were processed with a custom R script: ControlType weights were set to 0, spots with background channel more than 50 above the test channel were set to the median background intensity, background correction was performed with limma::backgroundCorrect() using the ‘normexp' method and an offset of 50, limma::normalizeWithinArrays() with the ‘loess' method, and finally limma::normalizeBetweenArrays() with the ‘Aquantile' method (all from the limma Bioconductor package). Finally, gene expression values were quantified as ‘expression ratio', the ratio of the normalized signals in the test and reference channels. For both platforms, the probeset (Affymetrix) or probe (Agilent) with highest interquartile range (IQR) across all samples (without regard to sample meta-data) was selected for further analysis, and used as the ‘official' level of the gene in that data set^46^. For all microarray data, differential expression analysis was performed with the limma package^47^.

### Heat maps

For Fig. 5, the count data were first transformed using the ‘regularized log transformation' (rlogTransformation function from DESeq2). After this, the 2.5% most variable genes were selected (by standard deviation, without regard to sample meta-data) and used for the heat-map. Hierarchical clustering was performed using the hclust function, with the ‘ward.D' clustering method and Euclidean distance function. For the heat maps in Figs 1, 2 and 9, nRPKM values were simply log2-transformed, ‘floored' at −4 (that is, any log2(nRPKM) value below −4 was set to −4), then *Z*-score transformed within each data set. In Fig. 9, all *Z*-scores greater than 4 were coloured at 4.00 for visualization purposes. For microarrays, log2-scale probeset or probe intensities were *Z*-score transformed. The selected genes in each plot were those differentially expressed at adjusted *P*-value≤0.05 and log2-fold change ⩾2, unless otherwise noted. As necessary, orthologues^48^ were displayed in subsequent data sets.

### Triangle plots

The triangle plots in Figs 1, 2 and 9 were created starting with the three-dimensional vector of the mean nRPKM of each gene in each of the three cell types. Then these means were normalized to 1, so that they all lay within the plane *x*+*y*+*z*=1. The representation shows their position on the triangle, which is the intersection of this plane with the first octant. This is achieved by multiplication with the matrix [ [−1 1 0] [ 0 0 sqrt(3)] ].

## Additional information

**Accession codes:** RNA-Seq and microarray data have been deposited to NCBI GEO (www.ncbi.nlm.nih.gov/geo/) and are available as accession numbers GSE75246 (LPS study RNA-Seq), GSE74995 (PS2APP bulk cortex microarray), GSE75357 (PS2APP bulk cortex RNA-Seq) and GSE75431 (PS2APP sorted cells RNA-Seq).

**How to cite this article:** Srinivasan, K. *et al*. Untangling the brain's neuroinflammatory and neurodegenerative transcriptional responses. *Nat. Commun.* 7:11295 doi: 10.1038/ncomms11295 (2016).

## Supplementary Material

Supplementary Information Supplementary Figures 1-6 and Supplementary Table 1

Supplementary Data 1 Comparison of cell type-enriched gene expression in this study to GSE52564. Our RNA-seq data from adult mouse brain neurons, astrocytes, and microglia was compared against recent data for the same cell types recovered from postnatal mouse brain (GSE52564). To explore the interactive plots and tables, download and unpackage the .zip file, and then open the index.html file in your browser (Firefox recommended). If you use Safari or Chrome, the plots and tables will not be rendered unless you change the browser settings; instructions for how to do so are provided within the index.html file.

Supplementary Data 2 LPS-induced expression in astrocytes, microglia and neurons. The effects of peripheral endotoxemia on gene expression in brain microglia, astrocytes, and neurons are displayed in interactive plots and tables derived from RNA-Seq data. Interactive comparisons of the endotoxemia response between each pair of cell types are also provided. To explore this dataset, download and unpackage the .zip file, and then open the index.html file in your browser (Firefox recommended). If you use Safari or Chrome, the interactive plots and tables will not be rendered unless you change the browser settings; instructions for how to do so are provided within the index.html file.

Supplementary Data 3 LPS-induced RNA processing in astrocytes, microglia and neurons. The effects of peripheral endotoxemia on alternative RNA processing events in brain microglia, astrocytes, and neurons are displayed in interactive plots and tables derived from RNA-Seq data. Comparisons of these effects between each pair of cell types are also provided. To explore these interactive plots and tables, download and unpackage the .zip file, and then open the index.html file in your browser (Firefox recommended). If you use Safari or Chrome, the plots and tables will not be rendered unless you change the browser settings; instructions for how to do so are provided within the index.html file.

Supplementary Data 4 Comparison of RNA-Seq and Fluidigm splicing assays. Selected LPS-induced RNA processing events identified by RNA-Seq in one cohort were retested in another cohort by quantitative PCR using customdesigned primers and the Fluidigm platform. Note that data for all three cell types per event are displayed, even though for some events only one or two cell types were being tested for validation of the RNA-Seq data (see Figure 7e). To explore these interactive plots and tables, download and unpackage the .zip file, and then open the index.html file in your browser (Firefox recommended). If you use Safari or Chrome, the plots and tables will not be rendered unless you change the browser settings; instructions for how to do so are provided within the index.html file.

Supplementary Data 5 NCBIM37 coordinates of mouse gene models for RNASeq analysis. Gene coordinates used in our RNA-Seq data analysis pipeline are provided, using version mm9 of the mouse genome and gene models from our internal database called IGIS (mostly based on RefSeq).

Supplementary Data 6 Comparison of HTSeqGenie/nRPKM analysis to Tophat/Cufflinks/FPKM analysis. Using a publicly available RNA-Seq dataset of gene expression in different cell types recovered from postnatal mouse brain (GSE52564), we compared our gene expression levels, based on GSNAP and HTSeqGenie with nRPKM normalization, to those reported by the original authors, based on Tophat and Cufflinks with FPKM normalization. We find the two methods disagree slightly due to lack of size-factor normalization in the FPKM method. nRPKM normalization better reveals expected upregulation of myelin-associated genes in mature oligodendrocytes, and also results in better agreement with the same authors' published microarray data.

## References

[b1] Cardona A. E., Huang D., Sasse M. E. & Ransohoff R. M. Isolation of murine microglial cells for RNA analysis or flow cytometry. Nat. Protoc. 1, 1947–1951 (2006) . 17487181 10.1038/nprot.2006.327

[b2] Sedgwick J. D. . Isolation and direct characterization of resident microglial cells from the normal and inflamed central nervous system. Proc. Natl Acad. Sci. USA 88, 7438–7442 (1991) . 1651506 10.1073/pnas.88.16.7438 PMC52311

[b3] Zamanian J. L. . Genomic analysis of reactive astrogliosis. J. Neurosci. 32, 6391–6410 (2012) . 22553043 10.1523/JNEUROSCI.6221-11.2012 PMC3480225

[b4] Saxena A. . Trehalose-enhanced isolation of neuronal sub-types from adult mouse brain. BioTechniques 52, 381–385 (2012) . 22668417 10.2144/0000113878 PMC3696583

[b5] Huntley M. A., Bien-Ly N., Daneman R. & Watts R. J. Dissecting gene expression at the blood-brain barrier. Front. Neurosci. 8, 355 (2014) . 25414634 10.3389/fnins.2014.00355 PMC4222230

[b6] Robinson A. P., Rodgers J. M., Goings G. E. & Miller S. D. Characterization of oligodendroglial populations in mouse demyelinating disease using flow cytometry: clues for MS pathogenesis. PLoS ONE 9, e107649 (2014) . 25247590 10.1371/journal.pone.0107649 PMC4172589

[b7] Butovsky O. . Identification of a unique TGF-beta-dependent molecular and functional signature in microglia. Nat. Neurosci. 17, 131–143 (2014) . 24316888 10.1038/nn.3599 PMC4066672

[b8] Hickman S. E. . The microglial sensome revealed by direct RNA sequencing. Nature neuroscience 16, 1896–1905 (2013) . 24162652 10.1038/nn.3554 PMC3840123

[b9] Zhang Y. . An RNA-sequencing transcriptome and splicing database of glia, neurons, and vascular cells of the cerebral cortex. J. Neurosci. 34, 11929–11947 (2014) . 25186741 10.1523/JNEUROSCI.1860-14.2014 PMC4152602

[b10] Rogers J. T. . CX3CR1 deficiency leads to impairment of hippocampal cognitive function and synaptic plasticity. J. Neurosci. 31, 16241–16250 (2011) . 22072675 10.1523/JNEUROSCI.3667-11.2011 PMC3236509

[b11] Lee S. . CX3CR1 deficiency alters microglial activation and reduces beta-amyloid deposition in two Alzheimer's disease mouse models. Am. J. Pathol. 177, 2549–2562 (2010) . 20864679 10.2353/ajpath.2010.100265 PMC2966811

[b12] Guez-Barber D. . FACS purification of immunolabeled cell types from adult rat brain. J. Neurosci. Methods 203, 10–18 (2012) . 21911005 10.1016/j.jneumeth.2011.08.045 PMC3221768

[b13] Chen-Plotkin A. S. . Variations in the progranulin gene affect global gene expression in frontotemporal lobar degeneration. Hum. Mol. Genet. 17, 1349–1362 (2008) . 18223198 10.1093/hmg/ddn023 PMC2900863

[b14] Richards J. G. . PS2APP transgenic mice, coexpressing hPS2mut and hAPPswe, show age-related cognitive deficits associated with discrete brain amyloid deposition and inflammation. J. Neurosci. 23, 8989–9003 (2003) . 14523101 10.1523/JNEUROSCI.23-26-08989.2003 PMC6740398

[b15] Lerman B. J. . Deletion of galectin-3 exacerbates microglial activation and accelerates disease progression and demise in a SOD1(G93A) mouse model of amyotrophic lateral sclerosis. Brain Behav. 2, 563–575 (2012) . 23139902 10.1002/brb3.75 PMC3489809

[b16] Rabin S. J. . Sporadic ALS has compartment-specific aberrant exon splicing and altered cell-matrix adhesion biology. Hum. Mol. Genet. 19, 313–328 (2010) . 19864493 10.1093/hmg/ddp498 PMC2796893

[b17] Kamphuis W., Orre M., Kooijman L., Dahmen M. & Hol E. M. Differential cell proliferation in the cortex of the APPswePS1dE9 Alzheimer's disease mouse model. Glia 60, 615–629 (2012) . 22262260 10.1002/glia.22295

[b18] Marlatt M. W. . Proliferation in the Alzheimer hippocampus is due to microglia, not astroglia, and occurs at sites of amyloid deposition. Neural Plast. 2014, 693851 (2014) . 25215243 10.1155/2014/693851 PMC4157009

[b19] Banks W. A. & Erickson M. A. The blood-brain barrier and immune function and dysfunction. Neurobiol. Dis. 37, 26–32 (2010) . 19664708 10.1016/j.nbd.2009.07.031

[b20] Schedlowski M., Engler H. & Grigoleit J. S. Endotoxin-induced experimental systemic inflammation in humans: a model to disentangle immune-to-brain communication. Brain Behav. Immun. 35, 1–8 (2014) . 24491305 10.1016/j.bbi.2013.09.015

[b21] Rivest S. Molecular insights on the cerebral innate immune system. Brain Behav. Immun. 17, 13–19 (2003) . 12615045 10.1016/s0889-1591(02)00055-7

[b22] Kang H. J. . Spatio-temporal transcriptome of the human brain. Nature 478, 483–489 (2011) . 22031440 10.1038/nature10523 PMC3566780

[b23] Chiu I. M. . A neurodegeneration-specific gene-expression signature of acutely isolated microglia from an amyotrophic lateral sclerosis mouse model. Cell Rep. 4, 385–401 (2013) . 23850290 10.1016/j.celrep.2013.06.018 PMC4272581

[b24] Anthony K. & Gallo J. M. Aberrant RNA processing events in neurological disorders. Brain Res. 1338, 67–77 (2010) . 20226177 10.1016/j.brainres.2010.03.008

[b25] Goldstein L. D. SGSeq: Prediction, quantification and visualization of alternative transcript events from RNA-seq data. R package version 1.0.6 .

[b26] Anders S., Reyes A. & Huber W. Detecting differential usage of exons from RNA-seq data. Genome Res. 22, 2008–2017 (2012) . 22722343 10.1101/gr.133744.111 PMC3460195

[b27] Chan C. W. . A novel member of the SAF (scaffold attachment factor)-box protein family inhibits gene expression and induces apoptosis. Biochem. J. 407, 355–362 (2007) . 17630952 10.1042/BJ20070170 PMC2275068

[b28] McCoy M. K. & Tansey M. G. TNF signaling inhibition in the CNS: implications for normal brain function and neurodegenerative disease. J. Neuroinflammation 5, 45 (2008) . 18925972 10.1186/1742-2094-5-45 PMC2577641

[b29] Park K. M. & Bowers W. J. Tumor necrosis factor-alpha mediated signaling in neuronal homeostasis and dysfunction. Cell. Signal. 22, 977–983 (2010) . 20096353 10.1016/j.cellsig.2010.01.010 PMC2860549

[b30] Nishitsuji K., Hosono T., Uchimura K. & Michikawa M. Lipoprotein lipase is a novel amyloid beta (Abeta)-binding protein that promotes glycosaminoglycan-dependent cellular uptake of Abeta in astrocytes. J. Biol. Chem. 286, 6393–6401 (2011) . 21177248 10.1074/jbc.M110.172106 PMC3057806

[b31] Ma Y. . Activated cyclin-dependent kinase 5 promotes microglial phagocytosis of fibrillar beta-amyloid by up-regulating lipoprotein lipase expression. Mol. Cell. Proteomics 12, 2833–2844 (2013) . 23816988 10.1074/mcp.M112.026864 PMC3790294

[b32] Ranganathan S. . LRAD3, a novel low-density lipoprotein receptor family member that modulates amyloid precursor protein trafficking. J. Neurosci. 31, 10836–10846 (2011) . 21795536 10.1523/JNEUROSCI.5065-10.2011 PMC3189500

[b33] Bien-Ly N., Gillespie A. K., Walker D., Yoon S. Y. & Huang Y. Reducing human apolipoprotein E levels attenuates age-dependent Abeta accumulation in mutant human amyloid precursor protein transgenic mice. J. Neurosci. 32, 4803–4811 (2012) . 22492035 10.1523/JNEUROSCI.0033-12.2012 PMC3433173

[b34] Drutel G. . Two splice variants of the hypoxia-inducible factor HIF-1alpha as potential dimerization partners of ARNT2 in neurons. Eur. J. Neurosci. 12, 3701–3708 (2000) . 11029639 10.1046/j.1460-9568.2000.00266.x

[b35] Sato F. . Functional analysis of the basic helix-loop-helix transcription factor DEC1 in circadian regulation. Interaction with BMAL1. Eur. J. Biochem. 271, 4409–4419 (2004) . 15560782 10.1111/j.1432-1033.2004.04379.x

[b36] Chung I. Y. & Benveniste E. N. Tumor necrosis factor-alpha production by astrocytes. Induction by lipopolysaccharide, IFN-gamma, and IL-1 beta. J. Immunol. 144, 2999–3007 (1990) . 2109008

[b37] Lieberman A. P., Pitha P. M., Shin H. S. & Shin M. L. Production of tumor necrosis factor and other cytokines by astrocytes stimulated with lipopolysaccharide or a neurotropic virus. Proc. Natl Acad. Sci. USA 86, 6348–6352 (1989) . 2474832 10.1073/pnas.86.16.6348 PMC297836

[b38] Robbins D. S. . Production of cytotoxic factor for oligodendrocytes by stimulated astrocytes. J. Immunol. 139, 2593–2597 (1987) . 3116087

[b39] Holm T. H., Draeby D. & Owens T. Microglia are required for astroglial Toll-like receptor 4 response and for optimal TLR2 and TLR3 response. Glia 60, 630–638 (2012) . 22271465 10.1002/glia.22296

[b40] Bien-Ly N. . Lack of widespread BBB Disruption in Alzheimer's Disease Models: Focus on Therapeutic Antibodies. Neuron 88, 289–297 (2015) . 26494278 10.1016/j.neuron.2015.09.036

[b41] Pau G. & Reeder J. HTSeqGenie: A NGS analysis pipeline. R package version 3.16.0 (2014) .

[b42] Wu T. D. & Nacu S. Fast and SNP-tolerant detection of complex variants and splicing in short reads. Bioinformatics 26, 873–881 (2010) . 20147302 10.1093/bioinformatics/btq057 PMC2844994

[b43] Love M. I., Huber W. & Anders S. Moderated estimation of fold change and dispersion for RNA-seq data with DESeq2. Genome Biol. 15, 550 (2014) . 25516281 10.1186/s13059-014-0550-8 PMC4302049

[b44] Untergasser A. . Primer3--new capabilities and interfaces. Nucleic Acids Res. 40, e115 (2012) . 22730293 10.1093/nar/gks596 PMC3424584

[b45] Irizarry R. A. . Exploration, normalization, and summaries of high density oligonucleotide array probe level data. Biostatistics 4, 249–264 (2003) . 12925520 10.1093/biostatistics/4.2.249

[b46] Bourgon R., Gentleman R. & Huber W. Independent filtering increases detection power for high-throughput experiments. Proc. Natl Acad. Sci. USA 107, 9546–9551 (2010) . 20460310 10.1073/pnas.0914005107 PMC2906865

[b47] Smyth G. K. in Bioinformatics and Compuational Biology Solutions using R and Bioconductor (Statistics for Biology and Health) eds Carey V., Gentleman R., Dudoit S., Irizarry R., Huber W. 397–420Springer (2005) .

[b48] NCBI_Coordinators. Database resources of the National Center for Biotechnology Information. Nucleic acids Res. 42, D7–17 (2014) . 24259429 10.1093/nar/gkt1146 PMC3965057

