# Supplementary material for: Untangling the brain's neuroinflammatory and neurodegenerative transcriptional responses
Source: Nat Commun. 2016 Apr 21;7:11295. doi: 10.1038/ncomms11295 (PMC4844685; doi:10.1038/ncomms11295)
Supplement: Supplementary Information — Supplementary Figures 1-6 and Supplementary Table 1 [file ncomms11295-s1.pdf]

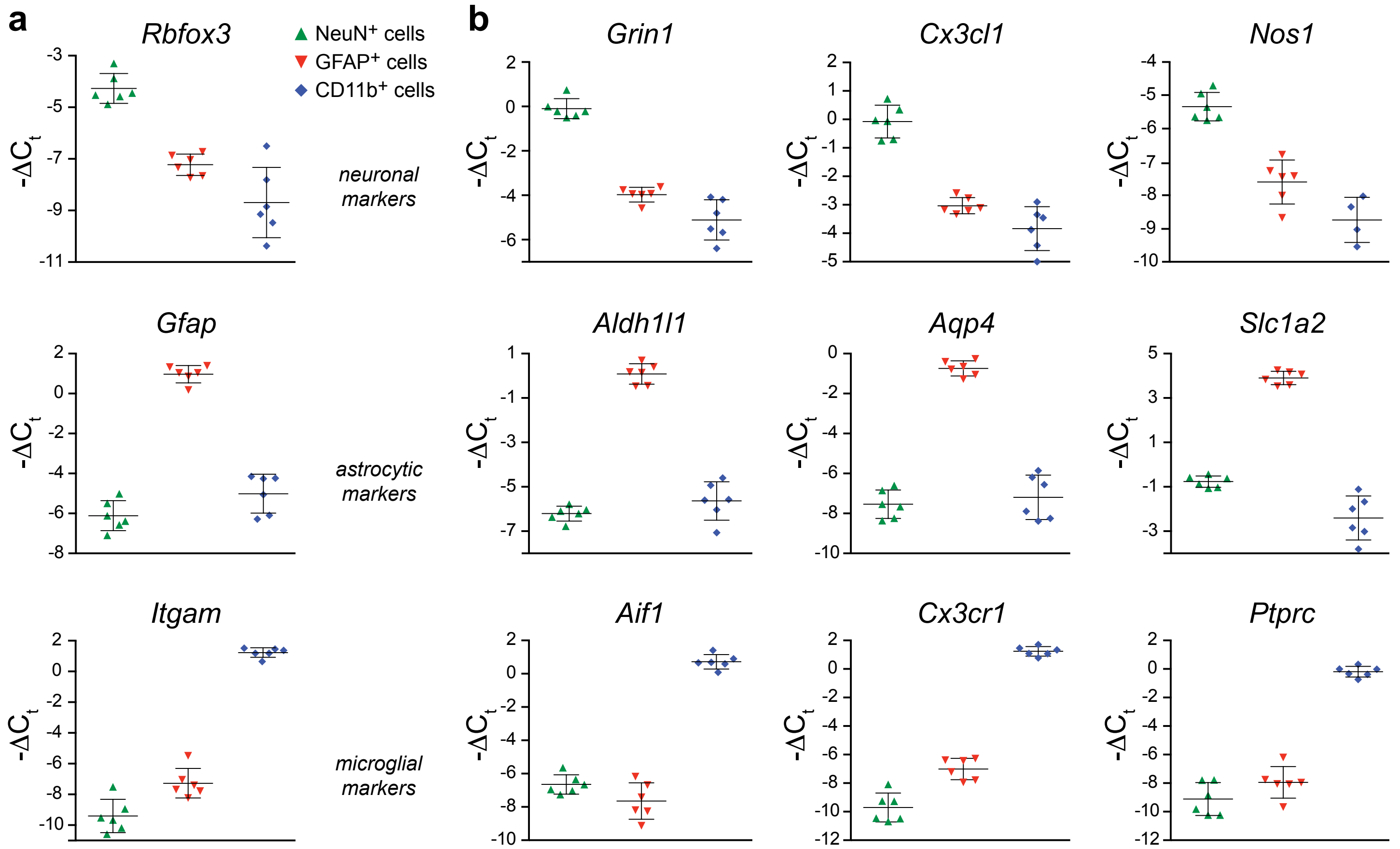

Supplementary Figure 1 | Cell type marker expression in FACS-isolated cells from adult mouse cortex (qPCR). (**a**) RNA from cells isolated using anti-NeuN (*Rbfox3*, neurons), anti-GFAP (astrocytes), or anti-CD11b (*Itgam*, microglia) was tested by RT-qPCR for expression of the same markers used for sorting. Markers were normalized to *Gapdh* expression, and bars represent mean ± s.d. (Prism). (**b**) Additional markers expressed by neurons (top row), astrocytes (middle row), and microglia (bottom row) were tested by RT-qPCR for expression level in NeuN$^+$, GFAP$^+$, and CD11b$^+$ sorted cell populations.

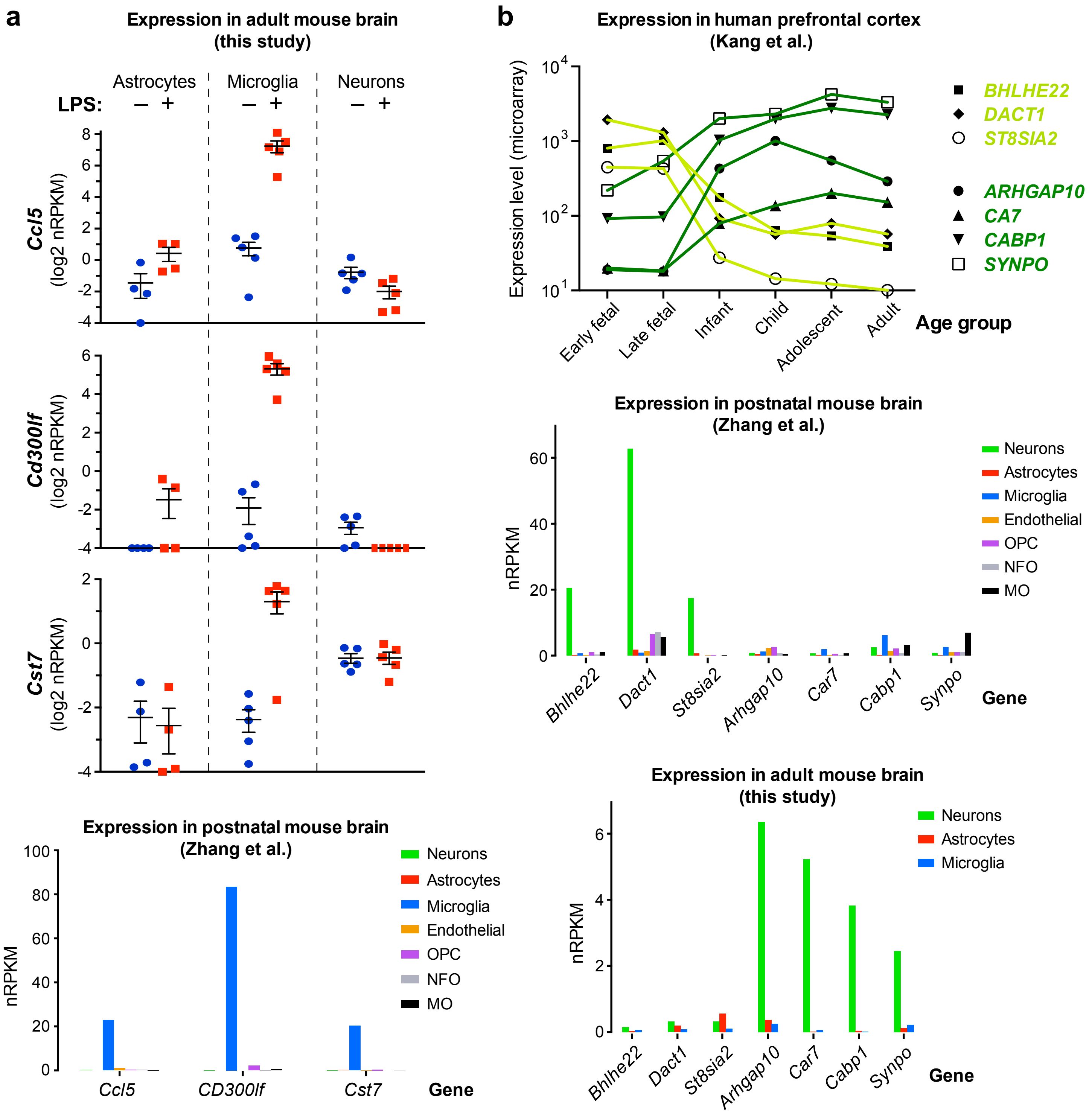

Supplementary Figure 2 | Our method yields expression profiles for microglia and neurons in their unperturbed, mature states. (**a**) Certain genes reported by others (Zhang et al., GSE52564) to be highly expressed in microglia (median values plotted, n=2 per cell type) were not observed in our data (GSE75246) unless the animals were somehow challenged as with LPS injection (n=4-5 samples per cell type plotted individually, bars = mean ± s.e.m. (Prism)). (**b**) Certain genes expressed in postnatal (GSE52564, n=2 per cell type) but not adult (GSE75246, n=4-5 per cell type) mouse neurons were robustly detected in fetal but not adult human prefrontal cortex (Kang et al., GSE25219). Conversely, certain genes expressed in adult but not postnatal mouse neurons were expressed more highly in adult than fetal human prefrontal cortex. Median values plotted. Early fetal = 8-17 weeks post-conception (n=88 tissue specimens), late fetal = 19-37 weeks post-conception (n=64), infant = < 1 year after birth (n=28), child = 1-9 years (n=32), adolescent = 11-19 years (n=28), and adult = 21-64 years (n=91). *Note: nRPKM values cannot be directly compared between GSE52564 and GSE75246 due to different library preparation methods. However, relative nRPKM differences among cell types within a study should be comparable between studies.

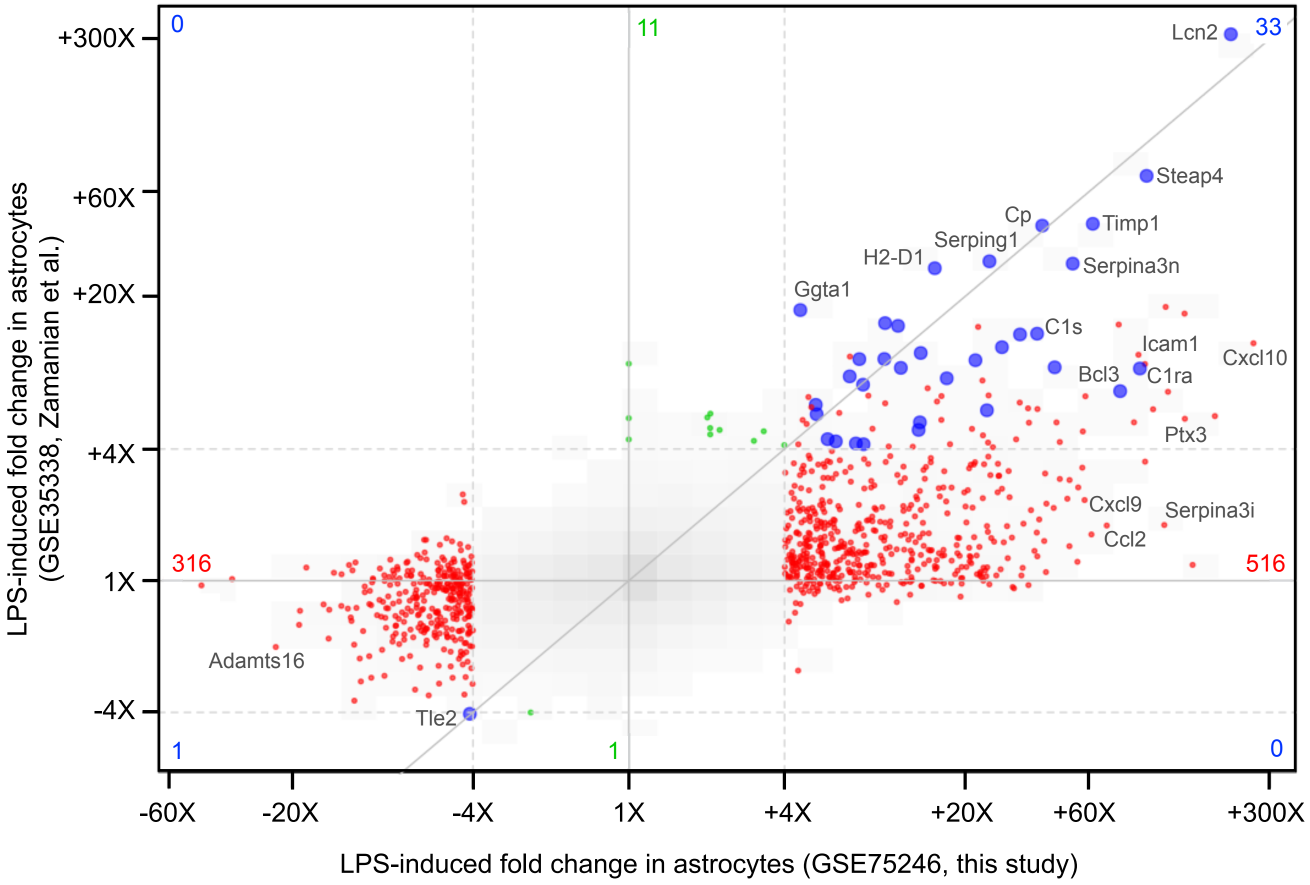

Supplementary Figure 3 | Comparison of expression profiles for astrocytic response to endotoxemia. This genome-wide "4-way" plot compares LPS-induced changes in astrocytic gene expression in this study (GSE75246) with those reported previously (GSE35338, Zamanian et al.). Each point represents a gene with ≥ 4-fold change (adjusted $p \leq 0.05$) in either study, with the fold-change between astrocytes from LPS-injected vs. control mice in our study plotted on the x-axis and that from GSE35338 on the y-axis. Blue points meet the cutoffs in both studies, red points only in our study, and green points only in GSE35338. The gray color indicates the distribution of fold-change values for all genes. (Genes for which no microarray data were available in GSE35338 are not plotted.) Possible reasons for the increased detection of LPS-induced genes in our study include the sensitivity of RNA sequencing compared to microarrays, the dose and strain of LPS used, and different methods for dissociating, labeling, and purifying astrocytes from intact mouse brain. See Supplementary Data 2 for interactive report of LPS-induced genes in our study.

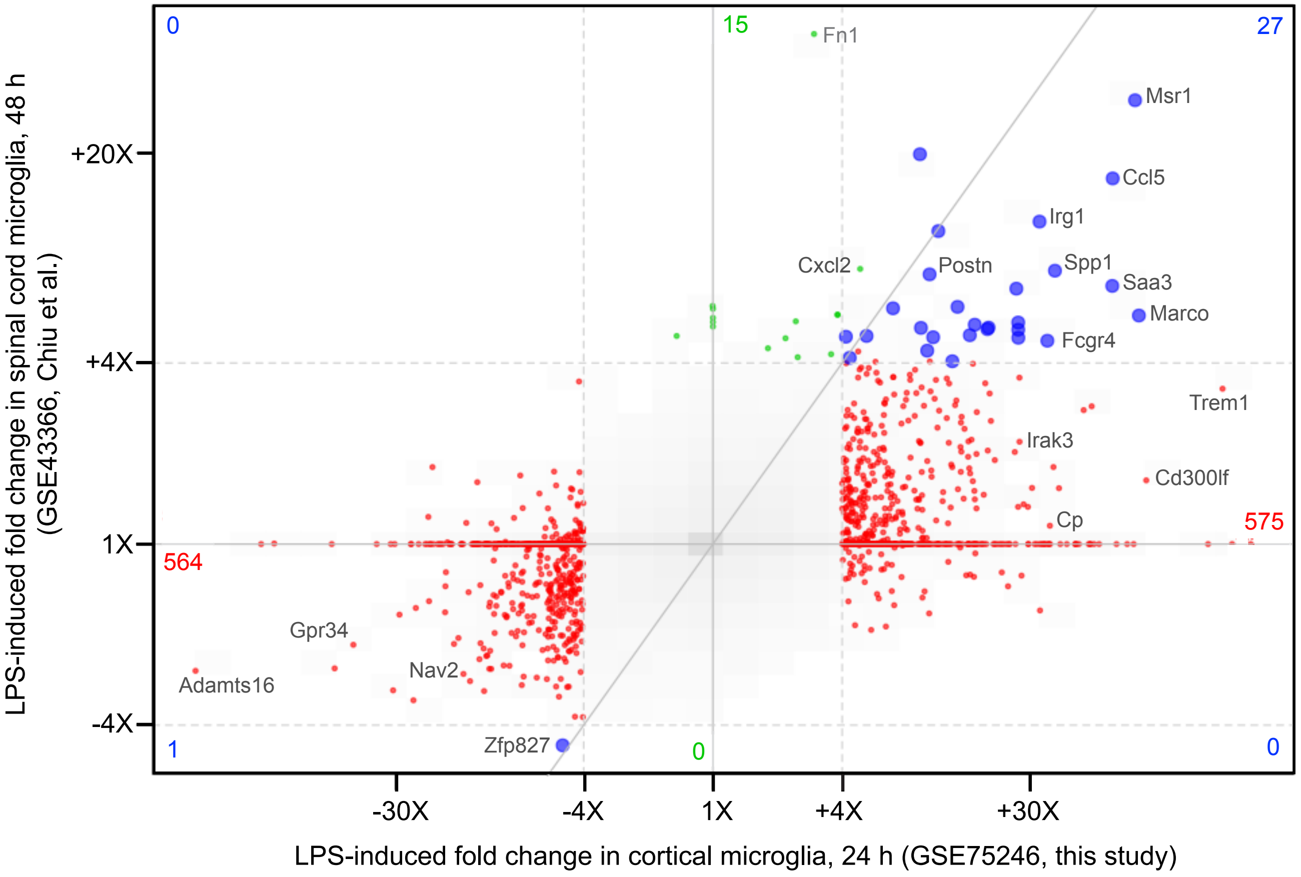

Supplementary Figure 4 | Comparison of expression profiles for microglial response to endotoxemia. This genome-wide "4-way" plot compares LPS-induced changes in microglial gene expression in this study (GSE75246) with those reported previously (GSE43366, Chiu et al.). Each point represents a gene with ≥ 4-fold change (adjusted $p \leq 0.05$) in either study, with the fold-change between microglia from LPS-injected vs. control mice in our study plotted on the x-axis and that from GSE43366 on the y-axis. Blue points meet the cutoffs in both studies, red points only in our study, and green points only in GSE43366. The gray color indicates the distribution of fold-change values for all genes. Possible reasons for the increased detection of LPS-induced genes in our study measuring the transcriptional response at an earlier time point post-injection, the dose and strain of LPS used, the larger sample size in our study, differences between brain and spinal cord, and the different methods for dissociating and purifying microglia from intact CNS tissue. See Supplementary Data 2 for interactive report of LPS-induced genes in our study.

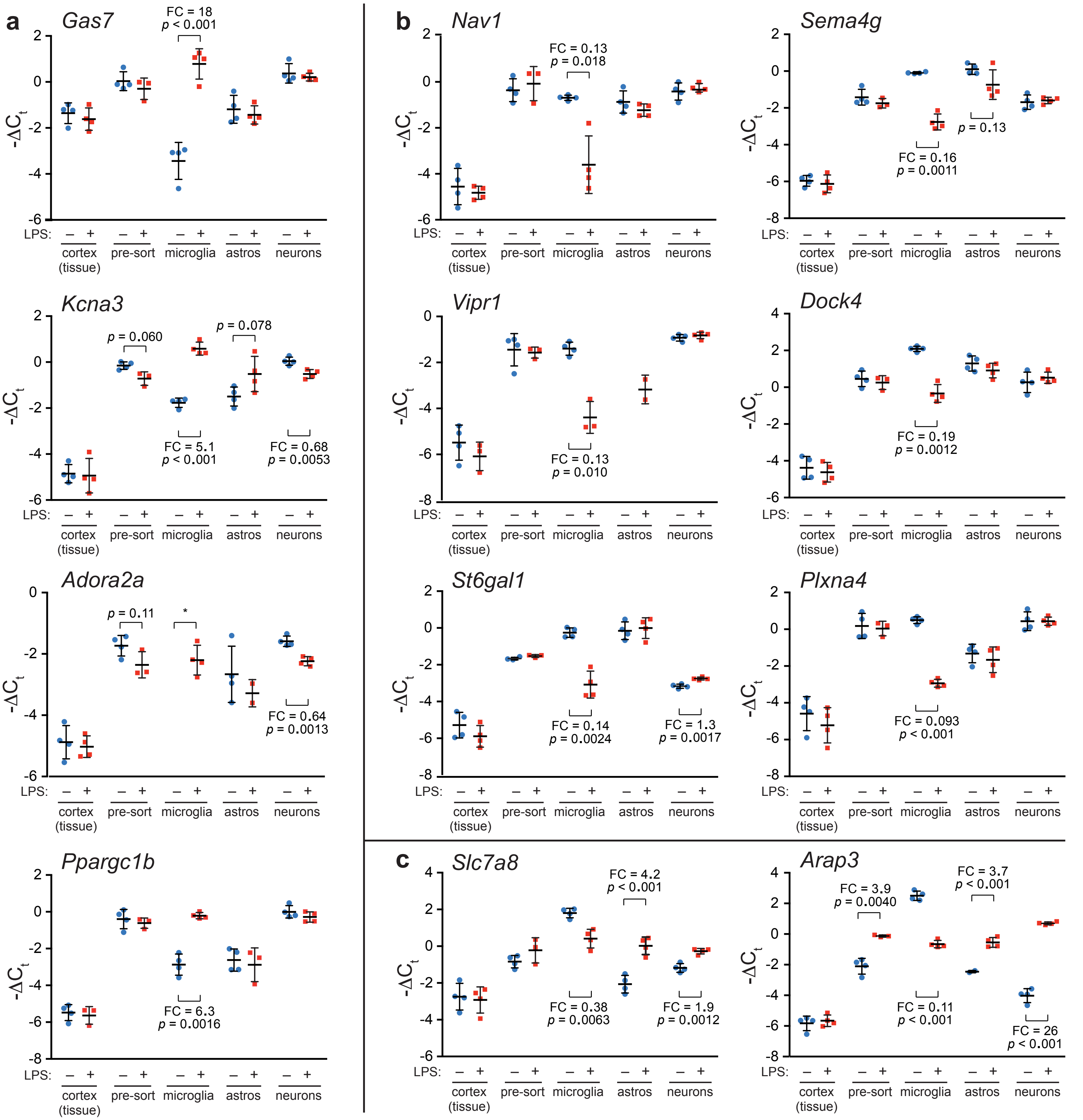

Supplementary Figure 5 | Our method allows detection of differentially expressed genes in purified cell types that cannot be detected using whole tissue RNA samples. 24 h after peripheral injections with LPS or saline (n=4/group), perfused mouse brains were processed using one cortical hemisphere for whole tissue RNA and the other for dissociating and purifying individual cell types, followed by RT-qPCR tests for LPS-induced changes. Genes such as *Gas7*, *Kcna3*, *Adora2a*, and *Ppargc1b* were upregulated (**a**) in microglia from LPS-injected mice, while genes such as *Nav1*, *Vipr1*, *St6gal1*, *Sema4g*, *Dock4*, and *Plxna4* were downregulated (**b**). These changes were not observed in RNA from whole tissue or from dissociated, DAPI$^+$ cells ("pre-sort") due to constitutive expression in other CNS cell types. *Slc7a8* and *Arap3* were LPS-induced in astrocytes and neurons, but these changes were not observed in whole tissue RNA due to offsetting decreases in microglial expression (**c**). Bars represent mean ± s.d., with fold change (FC) and unadjusted p-values (two-tailed t-test, unequal variance, unadjusted) shown. C_t values were normalized against *Gapdh* and *Hprt*. Note: For sorted cells, the fixation step affects RNA recovery for each gene differently, but consistently across samples for a given gene. Thus, although -ΔC_t values from whole brain (unfixed) are frequently different than pre-sort (fixed), ΔΔCt values (fold changes) are preserved when comparing across treatments or disease states. Data points and calculations for fold change and p-value are absent when the target gene was undetected. One LPS "pre-sort" sample failed to amplify in all assays.

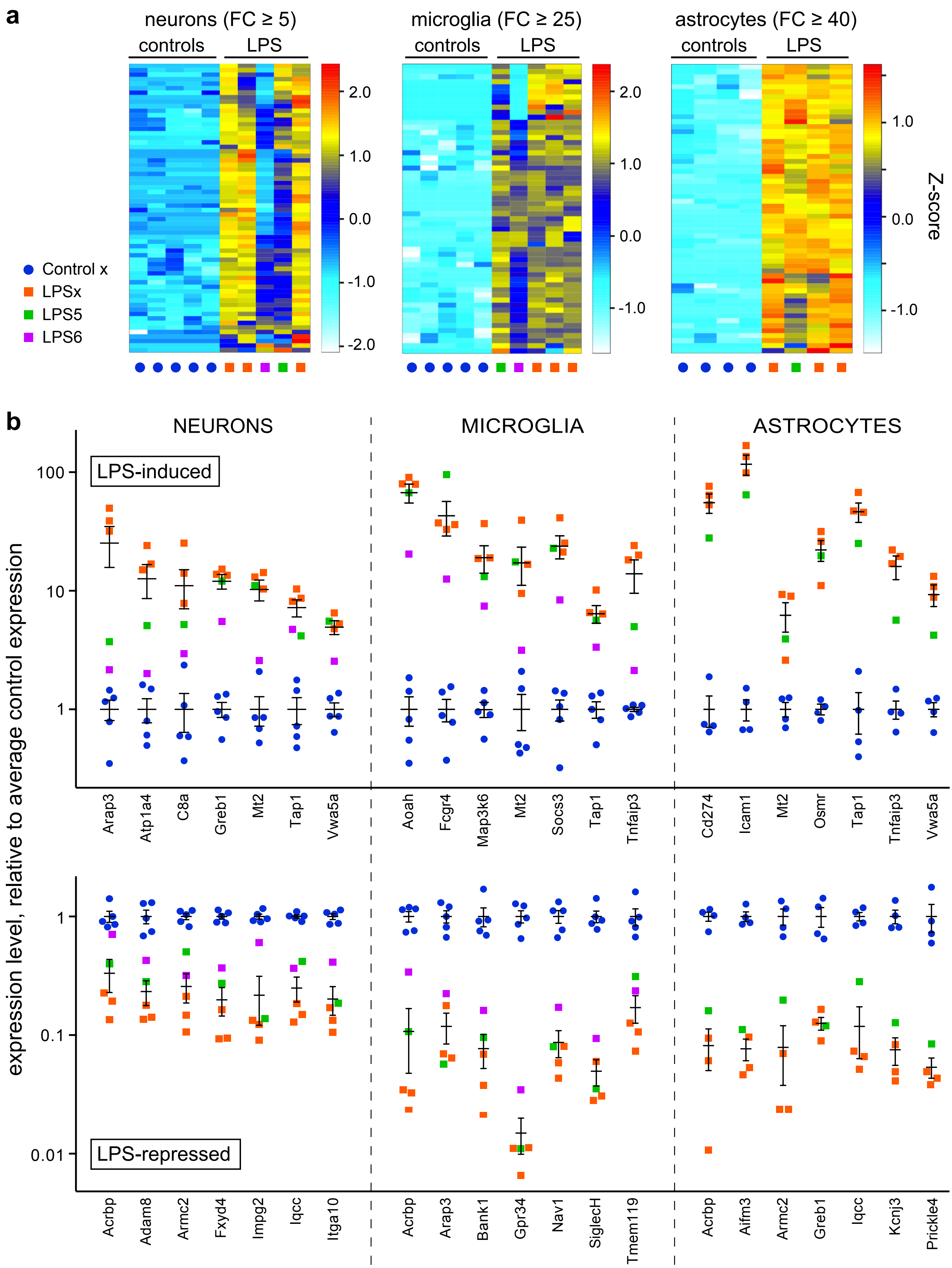
a
neurons (FC ≥ 5)
controls
LPS
microglia (FC ≥ 25)
controls
LPS
astrocytes (FC ≥ 40)
controls
LPS
Z-score
Control x
LPSx
LPS5
LPS6
b
NEURONS
MICROGLIA
ASTROCYTES
LPS-induced
LPS-repressed
expression level, relative to average control expression
Arap3
Atp1a4
C8a
Greb1
Mt2
Tap1
Vwa5a
Aoah
Fcgr4
Map3k6
Mt2
Socs3
Tap1
Tnfaip3
Cd274
Icam1
Mt2
Osmr
Tap1
Tnfaip3
Vwa5a
Acrbp
Adam8
Armc2
Fxyd4
Impg2
Iqcc
Itga10
Acrbp
Arap3
Bank1
Gpr34
Nav1
SiglecH
Tmem119
Acrbp
Aifm3
Armc2
Greb1
Iqcc
Kcnj3
Prickle4

Supplementary Figure 6 | Overall LPS response is consistent between cell types of a single animal. (**a**) For each cell type, a short list of the top ~60 LPS-induced genes was selected at $p \leq 0.05$ and the indicated fold-change (FC). Heat maps show the Z-score-normalized log2(nRPKMs) for these genes (rows) within the corresponding cell types, with each column representing that cell type from a single animal injected with either saline (blue dots) or LPS (colored squares). The LPS6 animal (magenta square) consistently showed a very weak transcriptional response in both neurons and microglia. (The astrocytes from this animal were excluded from the study due to neuronal contamination.) The LPS5 animal (green square) was also somewhat attenuated relative to the remaining three LPS-injected animals (orange squares). (**b**) Examples of LPS-induced (top chart) and LPS-repressed (bottom chart) gene expression from RNA-Seq data in each cell type demonstrate the overall trend that the transcriptional response in the LPS6 animal was severely attenuated in multiple cell types, and the response in the LPS5 animal was, overall, modestly attenuated in each cell type. Bars represent mean ± s.e.m. (Prism).

Gene	Event name	Event Type	Assay	ForwardOligo	ReverseOligo	DetectionOligo
Adarb1	110532_3_2/2_A5SS	A5SS	const	TCACCATGTCCTGCAGTGAC	ACGAAAATGCTGAGCAGGGA	ACGCTGGAACGTGGTGGGCATCCAGGGG
Adarb1	110532_3_2/2_A5SS	A5SS	incl	CCAGACTCTCACCAGCTGAC	TCCCCTCGCCAGACTCTATT	CCGAATCGCAAAGCAAGGGGACAGCTACGGACG
Adarb1	110532_3_2/2_A5SS	A5SS	skip	CGTGCTAGAGGAACCAGCAG	TCCCCTCGCCAGACTCTATT	CCGAATCGCAAAGCAAGGGGACAGCTACGGACG
Agap1	347722_1_2/2_SE	SE	const	GAGGACCACTGTGAAGGTGC	GACTGGAGATCGGTGCACAG	AAGAGGCCGCCCCGAGCCACGTCTGC
Agap1	347722_1_2/2_SE	SE	incl	GCAGTTTACACATCTCACCAAA	AAGGAGACCAGGCTGATGC	TGTCACTAGTGCGTCTGGGTCTCAGATGGCAAGCG
Agap1	347722_1_2/2_SE	SE	skip	CAAATTCAGACACGGGGCTG	GGTGGGTCGAGCTTGGGG	TCGGTGTGTTCCAGCCCAAGTATCTCCAGCAGCA
Akt3	23797_3_2/2_SE	SE	const	AGAGTGGACGGAAGCTATCC	TGTGAGGTTGGGCTACAATTCA	CCGTAGCCGACCGATTGCAGAGGCAAGAGGAGG
Akt3	23797_3_2/2_SE	SE	incl	AAATCACCCTTGGACTACTTTAAAAT	ACAATTCATCCTCTCCTCCTCT	CCAAGCCGTAGCCGACCGATTGCAGAGGCA
Akt3	23797_3_2/2_SE	SE	skip	GGAAAGAGAAGAGTGGACGGA	TGGGCTACAATTCATCCTCTCC	CCAAGCCGTAGCCGACCGATTGCAGAGGCA
Bag6	224727_6_2/2_SE	SE	const	GACATTCAGAGCCAGCGGAA	TTTCGTCTCTTGGCAGGCAT	ACCTCAGCCGCCCCTGAGTGATGCCTACCT
Bag6	224727_6_2/2_SE	SE	incl	GTGATGCCTACCTCAGTGGC	GCCTCTGAGAGTAGCAGCTG	CCAAGAGACGAAAGACAATGCAGGGTGAGGGCCCC
Bag6	224727_6_2/2_SE	SE	skip	GACGAAAGCTCCGGTCTGAT	CCCGATGGGCATTAGGGAAG	ACTGCAGGAAGATCCCAACTACAGCCCCCAGCG
Bap1	104416_1_2/2_SE	SE	const	AGCTCGAAGGGTCATCATGG	TGCCATCAGGTTGAAGCGAA	AGCGAATCGGTCTTGCTACTGCAGGGGAGCCC
Bap1	104416_1_2/2_SE	SE	incl	GGTGAACCGACAAACAGTCC	GTGGGTCTGAATCAGCTCCG	TGGAGGCCCTGCAGCAGCTGATTAGAGTAACCCAGC
Bap1	104416_1_2/2_SE	SE	skip	TGAGACCAGGCTACATGTACTG	CACCTCCTCTGCACCATCTG	AGGTGAACCGACAAACAGTCCTGGAGGCCCTGC
Brpf1	78783_1_2/2_A5SS	A5SS	const	CAAGCTCCTCAGTAGACCGG	CAGGGTAAACGTGGTCCCG	ACCGTGGCGAAAGTGCGTCTATACAGCGCCA
Brpf1	78783_1_2/2_A5SS	A5SS	incl	AGGCCAGAAGATCCAAAAGCT	CTGCCACTCCTGGTCCCG	CGGGAAACGCGAAGGTGCTTGCGGAATTGCG
Brpf1	78783_1_2/2_A5SS	A5SS	skip	GGGAAACGCGAAGATGTGAC	GGCTTAGTTGCCCGCAAATT	TGGGGGTGGACTTTGACGTGAAGACCTTCTGCCA
Caprin1	53872_1_2/2_SE	SE	const	ACTTGATGATTACCAGGAACGA	TCCAAATTATTTGTGACTTCCTGGT	AGGGGAAAGGCTCAATCAAGACCAGCTGGATGCCG
Caprin1	53872_1_2/2_SE	SE	incl	AGCATTTGAAGAGAACTGTTACAGA	TCCAAATTATTTGTGACTTCCTGGT	AGGGGAAAGGCTCAATCAAGACCAGCTGGATGCCG
Caprin1	53872_1_2/2_SE	SE	skip	GCTTCTCAGCATCCGGCAA	AGTTTACCCTTTTTCTTCTCCAGGT	CGGCGCCGTCCAGACCGAGGCCATGAAG
Cspp1	211660_6_2/2_SE	SE	const	GCCGAAATGAGGATCTACAACC	CCTTTGGCGTCTCTGAGAGG	CCTGGGGGAAGGGCGGAGGTGGTGCT
Cspp1	211660_6_2/2_SE	SE	incl	GAGGATCTACAACCCCTGGG	TCTGTCTGTGCATCCTATTCAA	AGGGCGGAGGTGGTGCTCCTCTCAGAGACGC
Cspp1	211660_6_2/2_SE	SE	skip	GAGGATCTACAACCCCTGGG	TGCAAAGGACTTATCAGATTTCCT	AGGGCGGAGGTGGTGCTCCTCTCAGAGACGC
Dmwd	13401_1_2/2_SE	SE	const	ATTGGACCCAGCTAAGGTGC	TTCTTGCACACGAGAGGCTC	TGTGCCCTCGGATCCATGAGGTGCCACTGC
Dmwd	13401_1_2/2_SE	SE	incl	CAAGAGGGCCTCATCTGCAC	AACTTGCTTGCCCTGCCT	AGGCGTTCACAGACGAGGAGACCGAGGCCC
Dmwd	13401_1_2/2_SE	SE	skip	CAAGGCGGGCATCTCCTC	GGGACATCCACATTTCACACC	CCAACCAGGCAGCTCCCCCAGTGGCACT
Dock7	67299_2_2/2_SE	SE	const	TGGGGATGATGCTTGCAACT	AAACGGTCTCCTTCCTGCTT	AGCTTCCGGCCTGCCACCCTCACTGTGG
Dock7	67299_2_2/2_SE	SE	incl	GAAGTCGAGATCAGTACTGGAG	GGTCAAGTTGCAAGCATCATCC	AGGCGGTCCCTGGAGAGGACGACCAGTGG
Dock7	67299_2_2/2_SE	SE	skip	TGATGCCACCAAGAACGGAA	GCTGGTCAAGTTGCAAGCAT	AGGCGGTCCCTGGAGAGGACGACCAGTGGG
Dzip1l	72507_1_2/2_SE	SE	const	ATAGCTACCACACGTGCCAC	GCATGTCTGCGTTGGATGT	ACAAGACCTTCATGAATGCCACCTTTCTCCGGGGCC
Dzip1l	72507_1_2/2_SE	SE	incl	ATGCAGGCATGGCAGATGT	ACTTGAGCTTGGCCCGAAG	AGCAGGAACAGCCACTGGGGGAGGTGCT
Dzip1l	72507_1_2/2_SE	SE	skip	GACAAGACCTTCATGAATGCCA	TCTCTAATTCCGACATCTGCCA	CCTTTCTCCGGGGCCACATCCAACGCAGACA
Epb4.9	13829_3_2/2_SE	SE	const	CAGGTGTTGGGCTACAAGGA	GTCAGGTCGCTCAATGTCCA	TCTGGCTGCCATCCCCAAGGACAAGGCCA
Epb4.9	13829_3_2/2_SE	SE	incl	AGCTCCTCCAGAGACTCCAG	CTTGTAGCCCAACACCTGGT	CCCGGCTCTCCCTCCAGCATCGTGGCCA
Epb4.9	13829_3_2/2_SE	SE	skip	CTGCAGAAGGCCAAGATGGA	ATGTCCAGGATGGCCTTGTC	TGTTGGGCTACAAGGATCTGGCTGCCATCCCCA
Esyt2	52635_5_2/2_SE	SE	const	ATCTCAGATGTCTGCGTCGC	CATCGACTCCCATGACTGGT	CACCGGTGGTGCCAACACTGCTCCGTCAAC
Esyt2	52635_5_2/2_SE	SE	incl	GGCGCCAGGACCTTGAGG	AGCAGTTGACTGAGTGGGAT	TGAGCAGCATCAGTGTTCTCTGGGGAGCCTGAGG
Esyt2	52635_5_2/2_SE	SE	skip	GAGGTTGAGGTCCTCCATCT	GCGACGCAGACATCTGAGAT	ACGGCCCTCTGTCTCCAAAGAAGGGAGGAAAATGCC
Fam172a	68675_2_2/2_SE	SE	const	GTGCAATCGAACTTGGTCGG	CAGATCTCTGGGCGAACTCC	CGCGGATCTTGAGAGAGAAAGTCCGAGCCAGGGCG
Fam172a	68675_2_2/2_SE	SE	incl	CAGAGATCTGGAAGGCCACG	AGACATCGATCTTCAGAAGACAGT	CCTCCTCGCACCTACCCTCTCAGCATTGCAGCT
Fam172a	68675_2_2/2_SE	SE	skip	CAGAGATCTGGAAGGCCACG	CAGCAAATCTGAGCTCTGTCC	CCTCCTCGCACCTACCCTCTCAGCATTGCAGCT
Fgd6	13998_1_2/2_SE	SE	const	GCGTTCTTTCATCCTCTCAGC	GGCATACTCTTCTATGGAGCTGG	CTGCCGCAGAAAGAGATGACTGGCTGGAGGCC
Fgd6	13998_1_2/2_SE	SE	incl	AGTATGCCAAGAAAAGAATCACATT	CCGAGGGGGCTAACTTCTTC	TGCCCCAGCAGGAGTCTTGACGAGGACTCAGAAAGG
Fgd6	13998_1_2/2_SE	SE	skip	GACGAGATTGTGTGCCAAGC	TCTGTAGCTCTTGGAAGCAGT	ACCTGAAAGGGCAACTGGCAAGAGTGTGCGAGC
Gphn	268566_8_2/2_SE	SE	const	TCGTTTCATCATTGGGGAATCC	ACCTGTTGTAACCCGCATCA	AGCTGGTGAGCAGCCAACTCAGACAGTAATGCCAGG
Gphn	268566_8_2/2_SE	SE	incl	AGATGATGGTACTGAAGAACTTGA	AATGTCATGGCCGATGGGT	AGTACGAATTCTGGTGCAAGCTCGGCCAGGCC
Gphn	268566_8_2/2_SE	SE	skip	TCAGATGATGGTACTGAAGAACTTGA	TCTTCAGGATTTAGCAGCGGA	AGTACGAATTCTGGTGCAAGCTCGGCCAGGCC
Htt	15194_13_2/2_SE	SE	E47-E48	ATTCTGCACTGCTGGAAGGT	GTCACCCGGTTCAGAATCAC	CAACCGTATCCCTGCTGAAGATATGAATGA
Htt	15194_13_2/2_SE	SE	E47-E52	ACTTCATGATGAGCTCGACCTC	CTGCAGGGATTCCACCATGT	AGTCATGTCACTTCGGCCTGCGAGATGGTGGC
Htt	15194_13_2/2_SE	SE	E52-E53	GAATCCCTGCAGTCAGTGCT	TCAGGAAACACTGTGCCAAA	GTGCTGAAGAACATTGTTATCAGTCTGGCC
Inpp5e	64436_1_2/2_SE	SE	const	GGACTTCAACTTCCGCCTGA	CAGCACATCCACCTCAGGTT	TGGTGGACGAGTGGCTGTGGAGGCCTTCC
Inpp5e	64436_1_2/2_SE	SE	incl	CCCGGAATGTGCCAGACA	CCCCAAACCAGAAGACCTCA	CCGCTCTAGTGCAGGGGATGTCACTACCCGGT
Inpp5e	64436_1_2/2_SE	SE	skip	TCACTTCACCTGGGATGTCAC	CCTCCACAGCCACTCGTC	TCTGGTTTGGGGACTTCAACTTCCGCCTGAGTGGTG
Kdm5a	214899_1_2/2_SE	SE	incl	TCCAATCTGGTGTCAGCCTT	GGAATATTCATTCTTGTTCCCCGC	TGAGGTTCTCAGCACTGACATCCAGCCTTCCCCAGA
Kdm5a	214899_1_2/2_SE	SE	skip	AGAAGAAATGGTCTAAAGTGGGT	AGAGTCTGACATAAGGCTGACAC	AGCCGCTTGGGATATCTGCCAGGAAAAGGAACTGGG
Larp4b	217980_3_2/2_SE	SE	const	ACCTCCAGTGTACAGTCCCC	GTGTTGTTGACCACGTCTGG	AGCAGCAGTTCCCTCTGTACAGCCTGATCACCCC
Larp4b	217980_3_2/2_SE	SE	incl	TCAGACAGTATCCTCCTAGAAGCA	TGCAGTGAAGTTAAATATTGAAGGACT	TGCGATTCCTAGTACAGAGAGAGGGCCTGGGCTGC
Larp4b	217980_3_2/2_SE	SE	skip	CAGTATCCTCCTAGAAGCAGGAAAA	GGGAAGTTGGACAGTCCCAG	CCAACACCACCAAAGCCTCCATCACCGAGCTTCG
Lsm14a	67070_2_2/2_SE	SE	const	CAGAAGCATCAAATAGTGGTACCT	TGTGGTTTTACAGATCTTGTGTCT	CCCAGAGCAATGCAGTTGGTTCGGCCTTCACACA
Lsm14a	67070_2_2/2_SE	SE	incl	AGCAATAGACAACCAAGAGCA	CCTCTTGTCATTTCTGAGTTGCT	CAGGCGAGCTGAAGTACACAAAGTCCCAAGGCCAGA
Lsm14a	67070_2_2/2_SE	SE	skip	TCCTTTGGAACAGAAGCATCA	TTGTGTACTTCAGCTTGGGC	CCCAGAGCAATGCAGTTGGTTCGGCCTTCACACA
Poldip3	73826_1_2/2_SE	SE	const	GGACTGAGCAGTTCCAAGCT	GGAGCGGTGTAGGCATCATT	CCATGTCCAAGGCCCTCCCTCTCACAAAAGTGGTCC
Poldip3	73826_1_2/2_SE	SE	incl	GTCAATAACCACCAGGCTAAACA	CATGTGGTGAACAAGGCTGC	TGCCTGTCCCTCCCAAGCAGATGAAGTTTGCAGCC
Poldip3	73826_1_2/2_SE	SE	skip	CTGCTGCGTTCACAAGCC	TCCAGGTCATACAAGTTCTGGA	CGCCCATTGGGACTGTAACCCCAGCTCTGAAGCTC
Polg	18975_1_2/2_SE	SE	const	GACTAGATGACCCTGCCTGG	CAGGTCAGTGCCATGAGCTT	CCCCAGCCTCCTCAGCCTGCAAATGCGG
Polg	18975_1_2/2_SE	SE	incl	GGGAGCTGGGCCCTTTGG	TAACCGGTTGTGGGCTGTT	CCCAGCGAGGAGGAGGAGCTTCAGCGAAGTGT
Polg	18975_1_2/2_SE	SE	skip	GGAGAGAGATCCTGGCCCG	CTCAGCTGCTGTAACCGGTT	CCCAGCGAGGAGGAGGAGCTTCAGCGAAGTGT
Sirpa	19261_2_2/2_S2E	S2E	const	GGGGATTCGACCGTTCTGAA	GGCCCTACTCCTCTGTACCA	TGCACTTTGACCTCCTTGTTGCCGGTGGGACCC
Sirpa	19261_2_2/2_S2E	S2E	incl	AACCATACCGTGCTGGGA	TCCAGTTGTGGGTAGCATTATT	TGCCCACTCGAGTGATCAAGGGAGCATGCAAACCT
Sirpa	19261_2_2/2_S2E	S2E	skip	TGTACTCGATAATAATGCTACCCACA	AGCAGGACTACGAGCAAAGC	TGGAATGTCTTCATCGGTGTGGGCGTGGCGT
Sltm	66660_7_2/2_SE	SE	const	GCTGAAAGGATTGCTCGGGA	GTGGGCGTTTCAAAGAATTCCT	AGAGAGGAGCTCAGAAGGCAGCAGCAGCAGCT
Sltm	66660_7_2/2_SE	SE	incl	GAACGCTTGGAAAGGGAACG	TCTCTCTCCCGAGCAATCCT	CGTATTGAACAGGAGCGGCGCAGGGAAGCTGA
Sltm	66660_7_2/2_SE	SE	skip	GGAATTCAGAAGGAGCGGCG	TGGGCGTTTCAAAGAATTCCT	AGAGAGGAGCTCAGAAGGCAGCAGCAGCAGCT
Tcf12	21406_1_2/2_SE	SE	const	GGACCTGCTAGAAGTGGCTG	AGCTCCTTGTCGGTCCCTAT	TGAATCCCCAGCAGCAGCGCATGGCCGC
Tcf12	21406_1_2/2_SE	SE	incl	GGTATGGATGAGCGTGGAGG	ACTGCTTCATCTCCTGTTTTGT	ACATCTTGGGGAACAAGTGGTCAACCAAGCCCCTCC
Tcf12	21406_1_2/2_SE	SE	skip	GGATGAGCGTGGAGGAACAA	GCTGTCTGTAAAACCTCTAGATGA	CATCTTGGGGAACAAGTGGTCAACCAAGCCCCTCCT
Usp16	74112_2_2/2_SE	SE	const	CTTTGTGTCAGCGTGCAGTC	CCGTGTCTGGAGCACTTCTC	AGTGCCAGCATGGGGAAGAAACGGACCAAGGG
Usp16	74112_2_2/2_SE	SE	incl	GAAACGGACCAAGGGGAGAA	CCTTTTCTAAGGTGTCTGCACA	TGCTCCAGACACGGTGGCCTCAGAGTCTGCA
Usp16	74112_2_2/2_SE	SE	skip	CTCAGAGTCTGCAGGGCTG	CGGATCTCGGTGTCGTGTAG	TGGCAGAGATTCTCAGGAGCAGCATGCCTTGAAGCA

SE = skipped exon

A5SS = alternative 5’ splice site

S2E = skipping 2 exons

Supplementary Table 1 | List of oligonucleotides used to detect alternatively processed RNA species induced in brain by peripheral endotoxemia (see Fig. 7 and Supplementary Data 4).
