# Supplementary material for: Untangling the brain's neuroinflammatory and neurodegenerative transcriptional responses
Source: Nat Commun. 2016 Apr 21;7:11295. doi: 10.1038/ncomms11295 (PMC4844685; doi:10.1038/ncomms11295)
Supplement: Supplementary Data 1 — Comparison of cell type-enriched gene expression in this study to GSE52564. Our RNA-seq data from adult mouse brain neurons, astrocytes, and microglia was compared against recent data for the same cell types recovered from postnatal mouse brain (GSE52564). To explore the interactive plots and tables, download and unpackage the .zip file, and then open the index.html file in your browser (Firefox recommended). If you use Safari or Chrome, the plots and tables will not be rendered unless you change the browser settings; instructions for how to do so are provided within the index.html file. [file ncomms11295-s2.zip › compareToZhangEtAl/index.html]

Supplementary Dataset 1. Comparison of Cell Type-Enriched Gene Expression in This Study to Zhang, et al


Table of Contents

- Supplementary Dataset 1. Comparison of Cell Type-Enriched Gene Expression in This Study to Zhang, et al
  - Comparison of cell type-enriched expression from this study and Zhang et al
    - cell.type: microglia - neuron
    - cell.type: astrocyte - microglia
    - cell.type: neuron - astrocyte
  - Heatmap

# Supplementary Dataset 1. Comparison of Cell Type-Enriched Gene Expression in This Study to Zhang, et al

RNA-Seq reads for project `GSE52564` were downloaded from NCBI and
analyzed by the same pipeline as our own data.
This was another study
in which the authors analyzed purified CNS cell types by RNA-Seq:

- Zhang *et al* Manuscript in *Journal of Neuroscience*
- GSE52564 Dataset in GEO

This report was generated with the AnalysisPageServer Bioconductor
package. For a guide to its interactive features, including
rollover, filtering, zoom, full-screen mode, and download, see
that package's vignette.

If you are opening this report from your own hard drive and the
plots and tables are
not rendering then local restrictions on your web browser may be preventing
it from accessing these data. This is called a "Local Deployment Error".
To turn off this restriction in Chrome it must be started with the
`--allow-file-access-from-files` switch. On a Mac open a Terminal and
type `open -a "Google Chrome" --args --allow-file-access-from-files`.
On windows Chrome can be started from the command line with
`"C:\PathTo\Chrome.exe" --allow-file-access-from-files`. (To find the
path to your Chrome executable open the URL chrome://version within
Chrome.) If data sets are not rendering in Safari, enable the
Developer menu (Preferences → Advanced → "Show Develop menu in menu
bar"), then select "Disable local file restrictions" from the Develop
menu.

## Comparison of cell type-enriched expression from this study and Zhang et al

Fold-change-fold-change
"4way" plots below compare cell-type differential for each pair of
cell types also present in our own data set. In each plot, every point
represents one gene. The x-axis indicates if the gene was identified
as differentially expressed in our own data, and the y-axis indicates
if it was differentially expressed in our analysis of *Zhang et al* data.
The following cutoffs were used to identify the most differentially expressed genes in both data sets:

- P-value: 0.05
- Fold-change: 20

The colors of the points indicate which genes were significantly
differentially expressed between the two cell types at these cutoffs:

- black: not significantly different in either data set
- red: significantly different only in the current study
- green: significantly different only in *Zhang et al*
- blue: significantly different both in this study and *Zhang et al* (including a very few genes with discordant changes)

### cell.type: microglia - neuron

### cell.type: astrocyte - microglia

### cell.type: neuron - astrocyte

## Heatmap

Another way to explore the similarity between samples is to look at a heatmap of expression.
Here we focus only on the 6186 genes which were significantly different between at least one pair
of cell types in at least one of the two datasets. Z-score normalization was performed within the data sets separately
for each of these genes.
In these heatmaps clustering reflects unsupervised
hierarchical clustering, and reveals that there is a good agreement in the profile of cell-type-enriched gene
expression as determined by both studies. "GSE75246" refers to this study, with samples labeled in green.
