# Supplementary figures and images for: Untangling the brain's neuroinflammatory and neurodegenerative transcriptional responses
Source: Nat Commun. 2016 Apr 21;7:11295. doi: 10.1038/ncomms11295 (PMC4844685; doi:10.1038/ncomms11295)

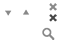

Supplement: Supplementary Data 1 — Comparison of cell type-enriched gene expression in this study to GSE52564. Our RNA-seq data from adult mouse brain neurons, astrocytes, and microglia was compared against recent data for the same cell types recovered from postnatal mouse brain (GSE52564). To explore the interactive plots and tables, download and unpackage the .zip file, and then open the index.html file in your browser (Firefox recommended). If you use Safari or Chrome, the plots and tables will not be rendered unless you change the browser settings; instructions for how to do so are provided within the index.html file. [file ncomms11295-s2.zip › compareToZhangEtAl/css/select2.png]

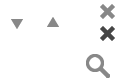

Supplement: Supplementary Data 1 — Comparison of cell type-enriched gene expression in this study to GSE52564. Our RNA-seq data from adult mouse brain neurons, astrocytes, and microglia was compared against recent data for the same cell types recovered from postnatal mouse brain (GSE52564). To explore the interactive plots and tables, download and unpackage the .zip file, and then open the index.html file in your browser (Firefox recommended). If you use Safari or Chrome, the plots and tables will not be rendered unless you change the browser settings; instructions for how to do so are provided within the index.html file. [file ncomms11295-s2.zip › compareToZhangEtAl/css/select2x2.png]

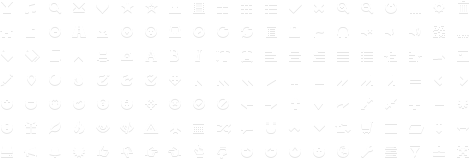

Supplement: Supplementary Data 1 — Comparison of cell type-enriched gene expression in this study to GSE52564. Our RNA-seq data from adult mouse brain neurons, astrocytes, and microglia was compared against recent data for the same cell types recovered from postnatal mouse brain (GSE52564). To explore the interactive plots and tables, download and unpackage the .zip file, and then open the index.html file in your browser (Firefox recommended). If you use Safari or Chrome, the plots and tables will not be rendered unless you change the browser settings; instructions for how to do so are provided within the index.html file. [file ncomms11295-s2.zip › compareToZhangEtAl/img/glyphicons-halflings-white.png]

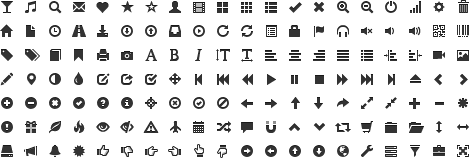

Supplement: Supplementary Data 1 — Comparison of cell type-enriched gene expression in this study to GSE52564. Our RNA-seq data from adult mouse brain neurons, astrocytes, and microglia was compared against recent data for the same cell types recovered from postnatal mouse brain (GSE52564). To explore the interactive plots and tables, download and unpackage the .zip file, and then open the index.html file in your browser (Firefox recommended). If you use Safari or Chrome, the plots and tables will not be rendered unless you change the browser settings; instructions for how to do so are provided within the index.html file. [file ncomms11295-s2.zip › compareToZhangEtAl/img/glyphicons-halflings.png]

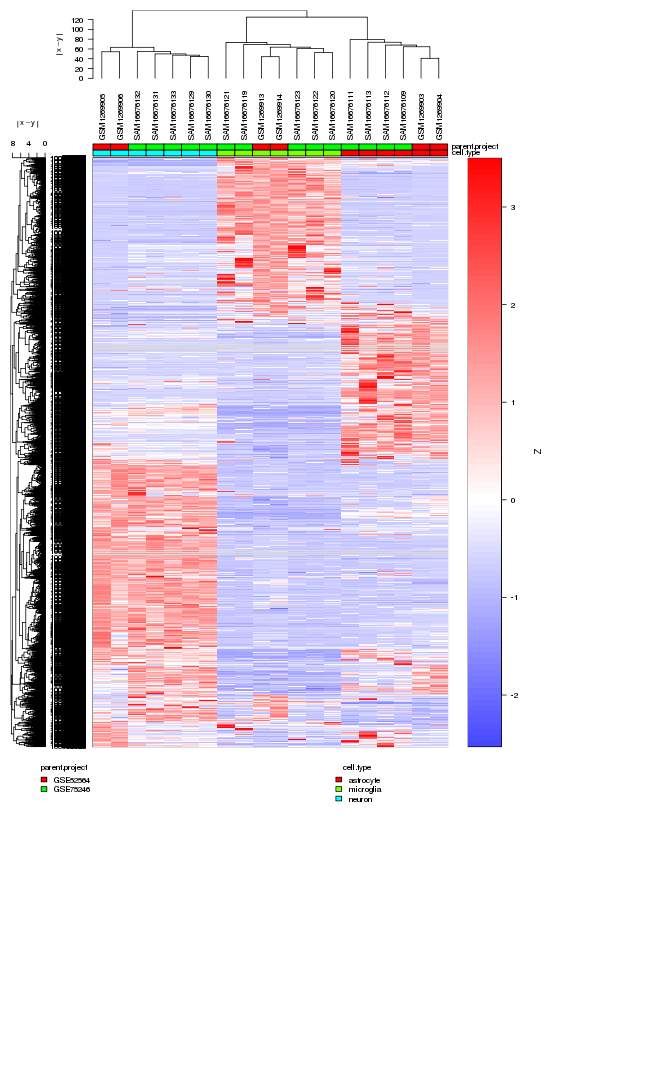

Supplement: Supplementary Data 1 — Comparison of cell type-enriched gene expression in this study to GSE52564. Our RNA-seq data from adult mouse brain neurons, astrocytes, and microglia was compared against recent data for the same cell types recovered from postnatal mouse brain (GSE52564). To explore the interactive plots and tables, download and unpackage the .zip file, and then open the index.html file in your browser (Firefox recommended). If you use Safari or Chrome, the plots and tables will not be rendered unless you change the browser settings; instructions for how to do so are provided within the index.html file. [file ncomms11295-s2.zip › compareToZhangEtAl/figure/unnamed-chunk-6-1.png]
