# Supplementary material for: Untangling the brain's neuroinflammatory and neurodegenerative transcriptional responses
Source: Nat Commun. 2016 Apr 21;7:11295. doi: 10.1038/ncomms11295 (PMC4844685; doi:10.1038/ncomms11295)
Supplement: Supplementary Data 2 — LPS-induced expression in astrocytes, microglia and neurons. The effects of peripheral endotoxemia on gene expression in brain microglia, astrocytes, and neurons are displayed in interactive plots and tables derived from RNA-Seq data. Interactive comparisons of the endotoxemia response between each pair of cell types are also provided. To explore this dataset, download and unpackage the .zip file, and then open the index.html file in your browser (Firefox recommended). If you use Safari or Chrome, the interactive plots and tables will not be rendered unless you change the browser settings; instructions for how to do so are provided within the index.html file. [file ncomms11295-s3.zip › lps-induction-dataset/index.html]

Supplementary Data Set 2: LPS-induced Expression in Astrocytes, Microglia and Neurons


Table of Contents

- Supplementary Data Set 2: LPS-induced Expression in Astrocytes, Microglia and Neurons
  - 2-way Plots
    - Astrocyte
    - Microglia
    - Neuron
  - 4-way Plots
    - Microglia/Neuron
    - Neuron/Astrocyte
    - Astrocyte/Microglia

# Supplementary Data Set 2: LPS-induced Expression in Astrocytes, Microglia and Neurons

As described in the main text, mice were treated with LPS or vehicle
control and sacrificed after 24 hours. Astrocytes, microglia and
neurons were FACS sorted and RNA was extracted from each cell type
population. RNA was then amplified and sequenced by RNA-Seq.

LPS-induced genes were detected in each cell type using the
DESeq2
Bioconductor package. The following cutoffs were used for this figure:

- Adjusted P-value: 0.05
- Fold-change: 4

This report was generated with the AnalysisPageServer Bioconductor
package. For a guide to its interactive features, including
rollover, filtering, zoom, full-screen mode, and download, see
that package's vignette.

If you are opening this report from your own hard drive and the
plots and tables are
not rendering then local restrictions on your web browser may be preventing
it from accessing these data. This is called a "Local Deployment Error".
To turn off this restriction in Chrome it must be started with the
`--allow-file-access-from-files` switch. On a Mac open a Terminal and
type `open -a "Google Chrome" --args --allow-file-access-from-files`.
On windows Chrome can be started from the command line with
`"C:\PathTo\Chrome.exe" --allow-file-access-from-files`. (To find the
path to your Chrome executable open the URL chrome://version within
Chrome.) If data sets are not rendering in Safari, enable the
Developer menu (Preferences → Advanced → "Show Develop menu in menu
bar"), then select "Disable local file restrictions" from the Develop
menu.

## 2-way Plots

These "2-way" plots compare expression (nRPKM values) between vehicle
(x-axis) and LPS (y-axis) mice for one cell type. Only genes meeting
both the P-value and fold-change cutoffs are activated in the plots. The
gray boxes indicate the background distribution of all genes in the
genome, including those which are not significant.

The tables below each plot show the following information:

| Column | Description |
| --- | --- |
| Feature Symbol | Feature Symbol |
| Feature Name | Feature Name |
| biotype | Biotype |
| log2(fold change) | Log2(Fold Change) |
| adjusted P-value | Adjusted P-Value |
| Low Expressed | Indicator for features omitted from analysis due to "low" counts |
| Group 1 Med. nRPKM | Median nRPKM for samples in first side of comparison |
| Group 2 Med. nRPKM | Median nRPKM for samples in second side of comparison |
| Mean Log2(nRPKM) | Mean Log2(nRPKM) for all samples in comparison |
| Group 1 Med. Count | Median raw count for samples in first side of comparison |
| Group 2 Med. Count | Median raw count for samples in second side of comparison |
| Cook's P-value | P-value for Maximum Cook's Distance. Significant values (close to 0) indicate a possible outlier. |
| Feature ID | Feature Identifier |
| chr | Chromosome |
| strand | Strand |
| start | Feature start coordinate |
| end | Feature end coordinate |

### Astrocyte

### Microglia

### Neuron

## 4-way Plots

The "4-way" plots compare LPS-induced gene expression in two different
cell types. As with the "2-way" plots each point corresponds to a
gene. The x-axis shows the LPS-induced fold-change of that gene in one cell
type, and the y-axis shows the fold-change in the other cell type.
Points further to the right or higher up correspond to genes
induced in the corresponding cells from LPS-treated
animals, whereas points further to the left or lower
down are repressed in cells from LPS-treated animals.

The color of the points indicate if their corresponding genes reached
the P-value
(0.05) and fold-change (4) cutoffs in one or both cell
types. Points corresponding which do not achieve these cutoffs in either cell type
are not activated in these plots. Similarly to the "2-way" plots, the
gray boxes indicate the background distribution of all genes in the
genome, including those which were not significantly differentially
expressed.

### Microglia/Neuron

### Neuron/Astrocyte

### Astrocyte/Microglia
