# Supplementary material for: Untangling the brain's neuroinflammatory and neurodegenerative transcriptional responses
Source: Nat Commun. 2016 Apr 21;7:11295. doi: 10.1038/ncomms11295 (PMC4844685; doi:10.1038/ncomms11295)
Supplement: Supplementary Data 3 — LPS-induced RNA processing in astrocytes, microglia and neurons. The effects of peripheral endotoxemia on alternative RNA processing events in brain microglia, astrocytes, and neurons are displayed in interactive plots and tables derived from RNA-Seq data. Comparisons of these effects between each pair of cell types are also provided. To explore these interactive plots and tables, download and unpackage the .zip file, and then open the index.html file in your browser (Firefox recommended). If you use Safari or Chrome, the plots and tables will not be rendered unless you change the browser settings; instructions for how to do so are provided within the index.html file. [file ncomms11295-s4.zip › LPS-induced-RNA-processing/index.html]

Supplementary Dataset 3. LPS-induced RNA Processing in Astrocytes, Microglia and Neurons


Table of Contents

- Supplementary Dataset 3. LPS-induced RNA Processing in Astrocytes, Microglia and Neurons
  - LPS-induced RNA Processing within each cell type ("2-way")
    - Astrocyte
    - Microglia
    - Neuron
  - Comparison of LPS-induced RNA Processing in different cell types ("4-way")
    - Astrocyte/Neuron
    - Neuron/Microglia
    - Microglia/Astrocyte

# Supplementary Dataset 3. LPS-induced RNA Processing in Astrocytes, Microglia and Neurons

The Bioconductor package `SGSeq` was run on each RNA-Seq sample.
SGSeq analyzes "transcript variants" of RNA processing "events". A transcript variant is what is often thought of as an alternative
isoform. For example, a "skipped exon" event would have two "transcript variants": one including the exon and one skipping it. Most
events have two transcript variants, although SGSeq can accommodate much more complicated structures.

For transcript variants with identical
5′ and 3′ features the counts from the two sides were averaged. For
transcript variants with different 5′ and 3′ features the counts were added.
For each variant, the 5′ and 3′ *total* counts were similarly combined.

The variants were then loaded into a `DEXSeqDataSet` object. Normally
`DEXSeq` looks for
changes in relative usage of the exons of a single gene, but here we used the same
statistical model to identify changes in relative usage of the variants of a single
RNA processing event. Finally, the first variant was discarded for each event and then
P-value-adjustment was performed.

The P-value and fold-change cutoff filters on this page are based on the results of
`DEXSeq`. The fold-changes refer to effect sizes in the statistical model. For the
purposes of visualization we present the variant frequencies in the two groups, which
are not the same thing as the effect sizes from `DEXSeq`. The variant frequencies are
what is called `PSI` ("percent spliced-in") by some authors. We also provide
the statistic `deltaVF`, which means "delta variant frequency": the
difference in variant frequencies between the two groups (called `ΔPSI` by
some authors). This statistic may be
more easily interpreted than the fold-change statistic.

`SGSeq` uses the following codes for the different types of splice events:

- `SE`: skipped exon (S: skip, I: inclusion)
- `S2E`: skipped two exons (S: skip, I: inclusion)
- `RI`: retained intron (E: exclusion, R: retention)
- `MXE`: mutually exclusive exons
- `A5SS`: alternative 5' splice site (P: proximal, D: distal)
- `A3SS`: alternative 3' splice site (P: proximal, D: distal)
- `AFE`: alternative first exon
- `ALE`: alternative last exon
- `AS`: alternative start other than AFE
- `AE`: alternative end other than ALE

Because our data had notable levels of intronic reads (we believe a
result of pre-mRNA enrichment by our isolation procedure and/or the
Nugen amplification method) we ignore the retained intron events.

- P-value: 0.05
- Fold-change: 2

This report was generated with the AnalysisPageServer Bioconductor
package. For a guide to its interactive features, including
rollover, filtering, zoom, full-screen mode, and download, see
that package's vignette.

If you are opening this report from your own hard drive and the
plots and tables are
not rendering then local restrictions on your web browser may be preventing
it from accessing these data. This is called a "Local Deployment Error".
To turn off this restriction in Chrome it must be started with the
`--allow-file-access-from-files` switch. On a Mac open a Terminal and
type `open -a "Google Chrome" --args --allow-file-access-from-files`.
On windows Chrome can be started from the command line with
`"C:\PathTo\Chrome.exe" --allow-file-access-from-files`. (To find the
path to your Chrome executable open the URL chrome://version within
Chrome.) If data sets are not rendering in Safari, enable the
Developer menu (Preferences → Advanced → "Show Develop menu in menu
bar"), then select "Disable local file restrictions" from the Develop
menu.

## LPS-induced RNA Processing within each cell type ("2-way")

These "2-way" plots compare, for one cell type, variant frequencies in vehicle-treated animals, on the
x-axis, to variant frequencies in LPS-treated animals, on the y-axis. The color indicates if the
difference between the two conditions reached our specified P-value
and fold-change cutoff. When a minimum number of reads (5) were not
detected in at least 3 samples of a given condition to
support either variant of a particular event the variant frequency was
set to 0.5 for visualization, which results in an excess of points
lining up along y=0.5 and x=0.5. On roll-over, or in the data table,
these points will be indicated with `medianVariantFreq` of NA.

The following meta-data are included for each
point:

| Field | Description |
| --- | --- |
| `log2FoldChange` | Log2 fold change from DEXSeq |
| `pvalue` | Raw P-value from DEXSeq |
| `padj` | Adjusted P-value from DEXSeq |
| `featureID` | Identifier for the transcript variant |
| `groupID` | Identifier for the event |
| `medianVariantFreq` | Median frequency for this variant for this cell type |
| `medianVariantFreq1` | Median frequency for this variant for vehicle-treated samples of this cell type |
| `medianVariantFreq2` | Median frequency for this variant for LPS-treated samples of this cell type |
| `medianVariantCount` | Median count for this variant for this cell type |
| `medianVariantCount1` | Median count for this variant for vehicle-treated samples of this cell type |
| `medianVariantCount2` | Median count for this variant for LPS-treated samples of this cell type |
| `medianTotalCount` | Median total count for all variants of this event for this cell type |
| `medianTotalCount1` | Median total count for all variants of this event vehicle-treated samples of this cell type |
| `medianTotalCount2` | Median total count for all variants of this event LPS-treated samples of this cell type |
| `deltaVF` | Changed in variant frequency between LPS and vehicle: `medianVariantFreq2 - medianVariantFreq1` |
| `chr` | Chromosome (mm9) |
| `start` | start coordinate of the event (mm9) |
| `end` | start coordinate of the event (mm9) |
| `strand` | strand of the event |
| `geneIDs` | Entrez gene ID(s) for this event (usually just 1, but could be more) |
| `geneSymbols` | Official gene symbols(s) for this event |
| `variantType` | Type of transcript variant (see above) |
| `event` | Link to UCSC genome browser for start-end region + 100 bp window |
| `junctions` | Coordinates of splice junctions (as last and first mm9 exonic bases) counted for the transcript variant. |
| `sig` | "Significant" if variant is significant LPS-induced (or repressed) at the given cutoffs, "Not" otherwise. |

### Astrocyte

### Microglia

### Neuron

## Comparison of LPS-induced RNA Processing in different cell types ("4-way")

These "4-way" plots compare LPS-induced RNA processing in two different
cell types. As with the "2-way" plots each point corresponds to a
transcript variant. The x-axis shows the LPS-induced `ΔVariantFreq` (that is, the
difference in variant frequency between LPS and vehicle treated samples) of that variant
in one cell type, and the y-axis shows the `ΔVariantFreq` in the other cell type.
Points further to the right or higher up correspond to RNA processing events with higher
inclusion rates in the corresponding cells from LPS-treated
animals, whereas points further to the left or lower
down correspond to events with lower inclusion in cells from LPS-treated animals.

The points shown are those corresponding to variants which were detected in both cell types.
The color of the points indicate if their corresponding genes reached
the P-value
(0.05) and fold-change (2) cutoffs in one (red or green) or both (blue) cell
types. Points corresponding to variants which do not achieve these cutoffs in either cell type
are shown in black in these plots.

### Astrocyte/Neuron

### Neuron/Microglia

### Microglia/Astrocyte
