# Supplementary material for: Untangling the brain's neuroinflammatory and neurodegenerative transcriptional responses
Source: Nat Commun. 2016 Apr 21;7:11295. doi: 10.1038/ncomms11295 (PMC4844685; doi:10.1038/ncomms11295)
Supplement: Supplementary Data 4 — Comparison of RNA-Seq and Fluidigm splicing assays. Selected LPS-induced RNA processing events identified by RNA-Seq in one cohort were retested in another cohort by quantitative PCR using customdesigned primers and the Fluidigm platform. Note that data for all three cell types per event are displayed, even though for some events only one or two cell types were being tested for validation of the RNA-Seq data (see Figure 7e). To explore these interactive plots and tables, download and unpackage the .zip file, and then open the index.html file in your browser (Firefox recommended). If you use Safari or Chrome, the plots and tables will not be rendered unless you change the browser settings; instructions for how to do so are provided within the index.html file. [file ncomms11295-s5.zip › splicing-RNASeq-fluidigm/index.html]

Supplementary Dataset 4: Comparison of RNA-Seq and Fluidigm Splicing Assays


Table of Contents

- Supplementary Dataset 4: Comparison of RNA-Seq and Fluidigm Splicing Assays
  - Gene-by-gene
    - Adarb1
    - Agap1
    - Akt3
    - Bag6
    - Bap1
    - Brpf1
    - Caprin1
    - Cspp1
    - Dmwd
    - Dock7
    - Dzip1l
    - Epb4.9
    - Esyt2
    - Fam172a
    - Fgd6
    - Gphn
    - Htt
    - Inpp5e
    - Kdm5a
    - Larp4b
    - Lsm14a
    - Poldip3
    - Polg
    - Sirpa
    - Sltm
    - Tcf12
    - Usp16
  - 4-way plots
    - microglia
    - neuron
    - astrocyte

# Supplementary Dataset 4: Comparison of RNA-Seq and Fluidigm Splicing Assays

A selection of 27 splice events identified as differentially spliced in response to LPS within at least one cell type
were analyzed by fluidigm qPCR. For each event, "skipping", "inclusion", and "constitutive" assays
were designed. The "skipping" assay has one primer on the skipping splice junction, with the other
either upstream or downstream. The "inclusion" assay has one primer completely within the included
region, with the other primer either upstream or downstream. The "constitutive" assay targets a
constitutively spliced region within 300 bases either upstream or downstream of, but not flanking,
the alternative event. Most of the events are cassette exons,
but in a few cases this definition was extended for other types of events. For example, for
an A3SS, the "skipping" junction would be the splice junction going to the downstream 3' splice site,
and the "inclusion" region would be the alternatively spliced region between the two 3' splice sites (called
the "extension" region in some splicing literature).

To avoid competition for the same cDNA, the three assay types were pre-amplified
in separate reaction, then run on separate chips. The control assay for Actb was included in all three
pools. For whole brain RNA, since no pre-amplification was required,
all the assays were run on a single chip. (Actb was still used as a
control assay.)

Passing Ct from technical replicates were averaged, and if no passing data
were available then the value was imputed as 1 plus the highest observed passing Ct for that
assay for any sample. (In the plots below imputed values are indicated with a distinct plotting symbol.) ΔCt
values were then calculated for each assay relative to the Actb loading control as

- ΔCtSplicing Assay = CtSplicing Assay - CtActb,

where "Splicing Assay" is "Incl", "Skip" or "Const".

Note that the three assays may have had slightly different values for their corresponding Actb
loading controls, since that assay was repeated on all chips. Finally, ΔΔCt
value were calculated for each splice event:

- ΔΔCt = ΔCtIncl - ΔCtSkip

With this convention for the sign, the negatives of this statistic, -ΔΔCt, has
greater values when the inclusion isoform is relatively more abundant, and
lesser values when the skipping isoform is relatively more abundant. This statistic then
corresponds to the ΔVF (ΔVariant Frequency) statistic derived from the `SGSeq` package.

This report was generated with the AnalysisPageServer Bioconductor
package. For a guide to its interactive features, including
rollover, filtering, zoom, full-screen mode, and download, see
that package's vignette.

If you are opening this report from your own hard drive and the
plots and tables are
not rendering then local restrictions on your web browser may be preventing
it from accessing these data. This is called a "Local Deployment Error".
To turn off this restriction in Chrome it must be started with the
`--allow-file-access-from-files` switch. On a Mac open a Terminal and
type `open -a "Google Chrome" --args --allow-file-access-from-files`.
On windows Chrome can be started from the command line with
`"C:\PathTo\Chrome.exe" --allow-file-access-from-files`. (To find the
path to your Chrome executable open the URL chrome://version within
Chrome.) If data sets are not rendering in Safari, enable the
Developer menu (Preferences → Advanced → "Show Develop menu in menu
bar"), then select "Disable local file restrictions" from the Develop
menu.

## Gene-by-gene

### Adarb1

### Agap1

### Akt3

### Bag6

### Bap1

### Brpf1

### Caprin1

### Cspp1

### Dmwd

### Dock7

### Dzip1l

### Epb4.9

### Esyt2

### Fam172a

### Fgd6

### Gphn

### Htt

### Inpp5e

### Kdm5a

### Larp4b

### Lsm14a

### Poldip3

### Polg

### Sirpa

### Sltm

### Tcf12

### Usp16

## 4-way plots

These "4-way" plots compare LPS-induced RNA processing as assayed by RNA-Seq,
in the first cohort, to RNA processing as assayed by Fluidigm, in the second cohort.
Each point corresponds to a
transcript variant assayed by Fluidigm (only these 27 are visualized here).
The x-axis shows the fold-change as reported by `DEXSeq`. This is essentially the
ratio of `VariantFreq` for the inclusion relative to skipping isoforms, and therefore
differs from `ΔVariantFreq`, which is shown in some other data sets. The y-axis shows
the fold-change as measured by Fluidigm, -ΔΔCt.
Points further to the right or higher up correspond to RNA processing events with higher
inclusion rates in the first cohort, assayed by RNA-Seq, or in the second cohort,
assayed by qPCR, respectively. Points further to the left or down exhibited greater skipping in the
first or second cohorts, respectively.

The color of the points indicate if their corresponding transcript variants reached
the P-value
(0.05) and fold-change (2) cutoffs in one (red or green) or both (blue) cohorts.
Points corresponding to variants which do not achieve these cutoffs in either cohort
are shown in black in these plots.

The points along the line `x = 0` (no fold-change by RNA-Seq) correspond to transcripts variants which were
filtered out for low RNA-Seq expression. The nominal fold-change, were it to be calculated, would not be
exactly 0.

### microglia

### neuron

### astrocyte
