# Supplementary material for: Untangling the brain's neuroinflammatory and neurodegenerative transcriptional responses
Source: Nat Commun. 2016 Apr 21;7:11295. doi: 10.1038/ncomms11295 (PMC4844685; doi:10.1038/ncomms11295)
Supplement: Supplementary Data 6 — Comparison of HTSeqGenie/nRPKM analysis to Tophat/Cufflinks/FPKM analysis. Using a publicly available RNA-Seq dataset of gene expression in different cell types recovered from postnatal mouse brain (GSE52564), we compared our gene expression levels, based on GSNAP and HTSeqGenie with nRPKM normalization, to those reported by the original authors, based on Tophat and Cufflinks with FPKM normalization. We find the two methods disagree slightly due to lack of size-factor normalization in the FPKM method. nRPKM normalization better reveals expected upregulation of myelin-associated genes in mature oligodendrocytes, and also results in better agreement with the same authors' published microarray data. [file ncomms11295-s7.zip › ncomms11295-s7.html]

Supplementary Dataset 6:


# Supplementary Dataset 6:

# Comparison of TopHat/Cufflinks to Genentech Pipeline on Zhang et al Data

## Introduction: Internal and Published Analyses

We used our own nRPKM values generated from our pipeline,
starting from the FASTQ files in
GSE52564,
and aligning to the mm9 genome, as described in our Methods. We refer to this analysis as the *internal* analysis.

We also downloaded the author's FPKM table from their website at
http://web.stanford.edu/group/barres\_lab/barreslab\_rnaseq.xlsx. This
was based on the Tophat/Cufflinks pipeline, also using the mm9 genome. We
refer to this analysis as the *published* analysis.

The internal annotation included 27797 genes
and the published annotation included 22458
genes. Based on gene symbols, there were 22274 in common,
and we only compared gene expression values for these.

## 2-way plots

First we generated “2-way” plots, which compare (internal)
`nRPKM` values on the x-axis to (published) `FPKM` on the
y-axis for each gene (black dots). Differences could be due to different gene models, different
aligners (GSNAP versus TopHat), different ways of counting (HTSeqGenie
versus CuffLinks), and different ways of normalizing. In this dataset
each cell type was represented by two samples; the average of the two values is plotted.

We added two lines to the plots. One, in black, is the line `Pub = Int`
(“Published equals Internal”), which
shows where the points would be if the results of the two pipelines
were identical. The other, in pink, is a fit line `Pub = Int * f` for some
factor `f`. (This becomes `log2(Pub) = log2(Int) + log2(f)` on the log scale, so results
in a translation of the line `Pub = Int` rather than a rotation.) This `f` is the simply the median of `Pub/Int` for highly
expressed genes (specifically, `log2(Pub) + log2(Int) >= 5`). When `f` is close to 1
the two lines are very close,
and the two pipelines have very similar results. When `f` is far from
1, but the points are still falling along the pink line, then the two
pipelines differ by a constant. The most egregious examples are
myelinating oligodendrocytes and microglia, for which gene expression
values from the published analysis are typically half that of our
internal analysis.

The factors `f` by which the two
methods disagree can be explained almost perfectly by the fact that
the published analysis using Cufflinks did not include size-factor normalization
but the internal analysis did. The expected discrepancy from these two
approaches, if nothing else were different, would be exactly the amount by which
RPKMs have to be adjusted to get nRPKMs, which is proportional to the ratio of
size-factor to total analyzed reads. This agrees very well with the
observed discrepancy:

(The line of fit above is `y = x * m` where `m = median(y/x)`.)

Therefore, of all the ways in which the two methods *could* differ,
the only substantial difference is size-factor normalization.

## Example genes

To the extent that the two methods disagree, how do we know which one is
correct? Let's look at a few example genes.

First consider Apoe. The internal analysis reports that it most highly
expressed in microglia, but the published analysis says that it
slightly higher in astrocytes:

Microglia is one of the cell types that has a big difference after the
normalization, whereas astrocytes do not. So it is not surprising that
this gene is a lot higher in microglia in the internal analysis, but
it is still hard to know which analysis is correct.

Now look at these genes encoding myelin-associated
proteins:

Myelinating oligodendrocytes, like microglia, also have a big
normalization factor, but newly formed oligodendrocytes do not.
So it is not surprising that these genes (and
most genes) should have about 2X higher expression in the myelinating
oligodendrocytes in the internal analysis. In this case, however, we
would expect these genes to be expressed at higher
levels in myelinating than in newly formed oligodendrocytes. So the
internal results are more consistent with what is known about the
biology of these genes.

## Microarray Breaks the Tie in favor of Internal Analysis

Although the myelin-associated proteins point to the internal analysis
as more biologically meaningful, we would like to know if this
generalizes to more genes and cell types. In order to address this, we
considered each pair of cell types and calculated log-fold-changes
from the internal nRPKMs and published FPKMs. We then used GSE9566, an older
microarray dataset also from the Barres lab
published in J. Neurosci. This
dataset lacked microglia and endothelial cells, but for cell
type pairs not involving these two we could calculate
the corresponding log-fold-changes from GSE9566. This is an independent
dataset, generated from a
different cohort of mice, using a completely different gene expression
platform and analysis pipeline. While for any one gene the microarray
might give a different result, if one of the RNA-Seq analyses
(internal or published) gave log-fold-changes that had better global
agreement with microarray log-fold-changes, this would suggest that
that the expression values generated by that analysis method more
closely matched the actual relative RNA concentrations in the
different cell types.

As an example, consider the fold-changes between gene expression in
OPCs and myelinating oligodendrocytes. There are three sources of
fold-changes: from the internal RNA-Seq analysis, from the published
RNA-Seq analysis and from the microarray RNA-Seq analysis. In the following
plots each point shows the log-fold-changes for a gene in two of the
three analyses. If the two methods are in agreement then most of the
points will fall along the gray line, `y = x`. The pink line,
`y = x + f` gives a fit. If `f` (which here is on log scale) is close
to zero then the two methods agree. If `f` is large, either negative
or positive, then the methods do not agree well.

The first panel shows the agreement between the internal and published
log-fold-changes. Here `f` is about 1, which means that the
fold-changes are off by about 2-fold. The second panel compares the
published to the microarray. Again `f` is about 1, which means that
the microarray systematically differs from the published RNA-Seq analysis by about the
same amount! Finally, the last panel compares the internal to the
microarray. In this case `f` is close to zero. We interpret this to
mean that the size-factor-normalized expression values from our
internal analysis are probably more faithful
to the actual RNA concentrations than the published analysis.

Following are the plots for the rest of the cell type pairs. In all
cases where the internal and published results differ (large `f` in
the first plot), the internal results show a better agreement with the
microarray data (`f` still large in the second plot but close to 0 in
the third plot).
